# A Review on Magnetic Induction Spectroscopy Potential for Fetal Acidosis Examination

**DOI:** 10.3390/s22041334

**Published:** 2022-02-10

**Authors:** Siti Fatimah Abdul Halim, Zulkarnay Zakaria, Jaysuman Pusppanathan, Anas Mohd Noor, Ahmad Nasrul Norali, Mohd Hafiz Fazalul Rahiman, Siti Zarina Mohd Muji, Ruzairi Abdul Rahim, Engku Ismail Engku-Husna, Muhamad Khairul Ali Hassan, Muhammad Juhairi Aziz Safar, Ahmad Faizal Salleh, Mohd Hanafi Mat Som

**Affiliations:** 1 Biomedical Electronic Engineering, Faculty of Electronic Engineering Technology, Universiti Malaysia Perlis, Arau 02600, Perlis, Malaysia; sitiabdulhalim@studentmail.unimap.edu.my (S.F.A.H.); anasnoor@unimap.edu.my (A.M.N.); ahmadnasrul@unimap.edu.my (A.N.N.); ahmadfaizal@unimap.edu.my (A.F.S.); mhanafi@unimap.edu.my (M.H.M.S.); 2 Medical Device & Life Sciences Cluster, Sports Engineering Research Centre (SERC), Universiti Malaysia Perlis, Arau 02600, Perlis, Malaysia; khairulhassan@unimap.edu.my (M.K.A.H.); juhairi@unimap.edu.my (M.J.A.S.); 3 Sport Innovation & Technology Centre (SiTC), Institute of Human Centered Engineering (iHumen), Universiti Teknologi Malaysia, Skudai 81310, Johor, Malaysia; jaysuman@utm.my; 4 Faculty of Electrical Engineering Technology, Universiti Malaysia Perlis, Arau 02600, Perlis, Malaysia; hafiz@unimap.edu.my; 5 Department of Electronic Engineering, Faculty of Electrical and Electronic Engineering, Universiti Tun Hussein Onn Malaysia, Parit Raja, Batu Pahat 86400, Johor, Malaysia; szarina@uthm.edu.my; 6 School of Electrical Engineering, Faculty of Engineering, Universiti Teknologi Malaysia, Skudai 81310, Johor, Malaysia; ruzairi@utm.my; 7 Department of Obstetrics and Gynaecology, School of Medical Sciences, Health Campus, Universiti Sains Malaysia, Kubang Kerian 16150, Kelantan, Malaysia; enhusna@usm.my

**Keywords:** fetal acidosis, fetal scalp, magnetic induction, spectroscopy

## Abstract

Fetal acidosis is one of the main concerns during labor. Currently, fetal blood sampling (FBS) has become the most accurate measurement of acidosis detection. However, it is invasive and does not provide a real time measurement due to laboratory procedures. Delays in diagnosis of acidosis have caused serious injury to the fetus, especially for the brain and the heart. This paper reviews the new technique in diagnosis of acidosis non-invasively. Magnetic Induction Spectroscopy (MIS) has been proposed to be a new device for acidosis detection in recent years. This paper explains the basic principle of MIS and outlines the design specifications and design considerations for a MIS pH probe. It is expected that readers will gain a basic understanding of the development of a MIS pH probe from this review.

## 1. Introduction

Maternal and child health are critical factors to consider during the labor and delivery process. Clinicians now routinely monitor fetal well-being and assess the risk of acidosis or associated sequelae [1]. The advancement of the medical field has resulted in the development of a variety of diagnostic techniques, both invasive and non-invasive, that can be used to determine the fetal state. It has evolved into the ultimate requirement for an obstetrician to assist them in investigating, making decisions, and appropriately treating the issue.

Fetal blood sampling (FBS) is a well-established technique for monitoring fetal acidosis with high precision. However, due to its invasive nature, bleeding and infection are the primary concerns [1,2]. Several non-invasive techniques such as a Doppler ultrasound, tocodynamometer, cardiotocogram, fetal pulse oximetry, and fetal electrocardiograph have been suggested to address these concerns. However, the low accuracy and precision of these methods result in faulty analysis, impairing decision-making, as evidenced by the rising number of unnecessary C-sections in recent years [3,4]. Current non-invasive techniques are insufficient and require significant design enhancements to meet standard requirements. Additionally, alternative methods for providing minimally invasive and more continuous monitoring devices should be explored to fulfill the clinical needs of intrapartum fetal monitoring [1].

This paper reviews the occurrence of fetal acidosis and describes the Magnetic Induction Spectroscopy (MIS) approach in fetal acidosis detection. The MIS technique is a novel alternative for non-invasive fetal monitoring that is based on fetal scalp pH measurement. The rest of this paper is structured as follows. Section 2 discusses the pH values of fetal blood and the current methods for determining fetal acidosis. Section 3 explains the theoretical concept of MIS and its application for biological tissues. Section 4 describes MIS probe design specifications for detecting acidosis. Section 5 details the suggested design considerations that should be made for future works. Finally, Section 6 draws the conclusions. It is hoped that this review will provide readers with a thorough understanding of MIS probe system design and a list of nomenclature used throughout this paper. 

## 2. Fetal Acidosis

Intrapartum or the transition period of labor is part of birth stress in which the fetus is compromised, receiving insufficient oxygen supply from placental circulation. It may create an extreme condition to the fetus either gradually or suddenly during the transition from maternal–fetal umbilical respiratory gas exchange to fetal lung activation [5]. Fetal acidosis or intrauterine hypoxia refers to a condition of deprived short supply of oxygen below the physiologic level of normal blood, which is defined as pH ≤ 7.25 [1,6,7]. 

### 2.1. Blood pH and Acidosis

The human body has a mechanism for regulating blood and extracellular fluid, which is called homeostasis. Homeostasis ensures that a biological system’s dynamic equilibrium is maintained in an optimal condition for survival. There are several types of homeostasis that occur in the blood circulatory system, including glucose, temperature, and pH. The pH value indicates the relative quantity of hydrogen ions ($H^{+}$) in a solution. It is defined as negative logarithm of $H^{+}$ concentration given by:

(1) $$\mathrm{pH}=-\log[H^{+}]$$

where [$H^{+}$] is hydrogen ion concentration in mol/L. The higher the $H^{+}\;$ concentration, the more acidic the solution and the lower the pH. A 10-fold change in concentration of $H^{+}$ is indicated by a single pH unit change [8]. Blood pH homeostasis is the principle that keeps blood pH relatively constant by buffering dissolved hydrogen ions in the blood due to cell respiration and metabolic activity. An imbalance of these two conditions will result in respiratory acidosis and metabolic acidosis [3]. 

Cell respiration can take place with or without oxygen, referred to as aerobic and anaerobic respiration, respectively. Respiratory acidosis is explained by aerobic cell respiration, which results in an increase in carbon dioxide partial pressure (p${\mathrm{CO}}_{2}$). ${\mathrm{CO}}_{2}$ will form carbonic acid ($H_{2}{\mathrm{CO}}_{3}$) when it comes into contact with water ($H_{2}O$). Carbonic acid is a weak acid that serves as a component of the blood buffering system for acid-base balance. Once a basic substance enters the bloodstream, carbonic acid immediately reacts with hydroxide ions and loose protons to form bicarbonate (${\mathrm{HCO}}_{3}{}^{-}$) and hydrogen ion ($H^{+}$). The carbonic acid–bicarbonate buffer system is represented by:

(2) $${\mathrm{CO}}_{2}+H_{2}O↔H_{2}{\mathrm{CO}}_{3}↔{\mathrm{HCO}}_{3}{}^{-}+H^{+}$$

The acidity of blood pH to the constituent bicarbonate buffering can be described by the Henderson–Hasselbalch equation:

(3) $$\mathrm{pH}={\mathrm{pK}}_{{\mathrm{aH}}_{2}{\mathrm{CO}}_{3}}+\log\frac{\left[{\mathrm{HCO}}_{3}{}^{-}\right]}{\left[H_{2}{\mathrm{CO}}_{3}\right]}$$

where ${\mathrm{pK}}_{\mathrm{aH}2\mathrm{CO}3}$ is the acid dissociation constant of carbonic acid, ${\mathrm{HCO}}_{3}{}^{-}$ is the concentration of bicarbonate in plasma and $H_{2}{\mathrm{CO}}_{3}$ is the concentration of carbonic acid in the blood. A lower ${\mathrm{pK}}_{a}$ value indicates a strong acidity of the blood as ${\mathrm{pK}}_{a}$ shows the tendency of an acid to dissociate into $H^{+}$ or the degree of ionization.

Metabolic acidosis can be explained by the accumulation of $H^{+}$ due to anaerobic respiration in glycolysis. Glycolysis is a process of energy production that takes place in both aerobic and anaerobic states. During glycolysis, glucose is broken down into pyruvate and energy. However, in anaerobic respiration, pyruvate is broken down into lactate and nicotinamide adenine dinucleotide (${\mathrm{NAD}}^{+}$). Lactate itself is broken down into lactic acid and $H^{+}$ ions [1] which reduces the pH and induces metabolic acidosis. Persistent metabolic acidosis may cause irreversible organ damage [3]. ${\mathrm{NAD}}^{+}$ is required for the limited energy production through glycolysis to continue.

(4) $$\mathrm{Pyruvate}\;\to \mathrm{Lactate}\;+{\mathrm{NAD}}^{+}$$

(5) $$\mathrm{Lactate}\;\to \mathrm{Lactic}\;\mathrm{Acid}+H^{+}$$

Overall, ${\mathrm{CO}}_{2}$ and lactate have contributed to the amount of $H^{+}$ found in the blood. If the amount $H^{+}$ exceeds the hemoglobin capacity it will affect carbonic acid equilibrium, thus reducing the blood pH. Maintaining an optimal pH balance is critical for the chemical reactions occurring in the body. The measurement of normal blood starts from pH > 7.25; pre-acidosis has a pH between 7.20 and 7.25, and acidosis occurs when pH < 7.20 as shown in Table 1 [1,6,9]. On the other hand, an increase in blood pH ≥ 7.45 is referred to as alkalosis. This measurement demonstrates the critical nature of maintaining an adequate oxygen supply in order to maintain an optimal pH value. The low oxygen in tissue causes acute and chronic hypoxia, which induces low oxygen in the blood called hypoxemia. This finally results in an acidosis condition (low blood pH) [10].

Deviation in systemic acidity requires physiological adaptations in the redistribution of blood flow to vital organs, most notably the heart and brain [5]. This can have a detrimental effect if fetal distress goes unmanaged, resulting in critical organ failure, particularly cerebral damage. Specifically, hypoxic–ischemic encephalopathy (HIE), cerebral palsy (loss of motor function; damage of white matter due to reduced blood or oxygen supply known as periventricular leukomalacia (PVL), which can be fatal when severe [7,11,12]. According to a previous study, out of 56574 babies born at 35 gestation weeks or more, 506 of them have pH ≤ 7 which is 0.9%, with three deaths occurring within an hour of birth and a total of 24 deaths before the age of 2 years [12].

Apart from pH, a few other parameters have been observed to change significantly during acidosis. In study [10], it was noticed that ${\mathrm{pO}}_{2}$, pH and bicarbonate concentrations decreased, whereas lactate and potassium concentrations increased. However, ${\mathrm{pO}}_{2}$ was the parameter that most quickly reflected changes in the acid-base status, reaching its minimum value within 2.38 min. It was followed by pH, which indicates the severity of an acidosis. Other metabolites (bicarbonate, potassium and lactate) exhibit a gradual change in response to the cellular metabolism, which induced a decrease in oxygen levels in the blood. Thus, ${\mathrm{pO}}_{2}$ becomes the predominant parameter for the onset of acidosis where ${\mathrm{pO}}_{2}\;$≤ 80 mmHg or ${\mathrm{SpO}}_{2}\;$≤ 95%. In addition, ${\mathrm{pO}}_{2}$ was the only metabolite capable of reaching a similar level in the basal period.

### 2.2. Current Fetal Acidosis Detection Method

Fetal acidosis is the most undesirable condition during labor; thus, monitoring is necessary in order to examine the health of the fetus by either an invasive or non-invasive method.

#### 2.2.1. Invasive Method

Electronic Fetal Monitoring (EFM), developed in the 1950s, introduced the cardiotocogram (CTG), which records the fetal heart rate and measures contraction frequency. The duration and strength of uterine contractions are related to the fetal heart rate (FHR) and its pattern. CTG consists of external devices (Doppler ultrasound and tocodynamometer), internal devices (intrauterine pressure catheters (IUPC) and fetal scalp electrodes (FSE)) [13]. A Doppler ultrasound provides an audible simulation of the fetal heartbeats, and the real-time audio sound is shared with everyone present in the room. Since the tocodynamometer provides an unreliable measure of contraction strength, it was replaced by IUPC with the evolution of the catheter-tip sensor. Nonetheless, this method is invasive in nature, and requires membrane rupture, which is one of the IUPC’s drawbacks [14]. CTG is very sensitive but not particularly specific, which means that a suspicious CTG signal can appear in the absence of any abnormality. As a result, it is necessary to increase the specificity, which is accomplished through pH.

**Figure 1:**
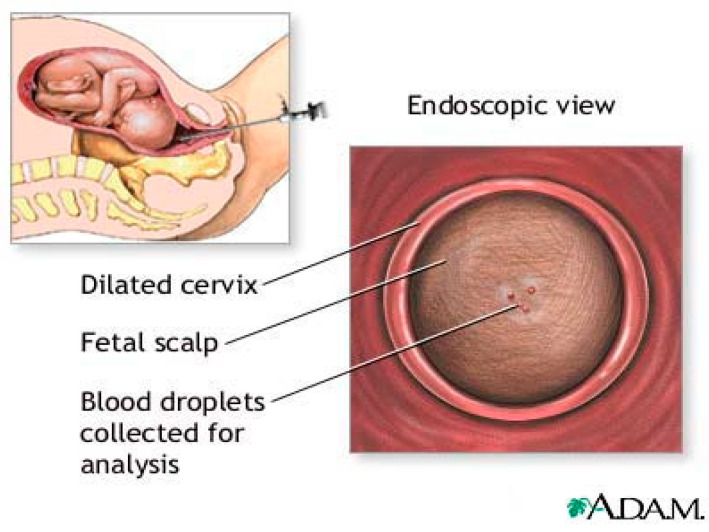
Fetal blood sampling. “Adapted with permission from Ref. [18], 2022, Jacobson, J.D.; Zieve, D.”.

In 1961, Hon introduced Fetal Blood Sampling (FBS) [3] as shown in Figure 1. It has long been recognized as the most accurate test for measurement of pH, ${\mathrm{pCO}}_{2}$ and base access. FBS is considered after CTG is pathological and considers expediting the birth [15]. FBS requires cervical dilation of 3–4 cm to accommodate a large amnioscope [3]. It is followed by a small scalpel incision to a depth of 1.5–2 mm [15] to obtain a blood sample using a thin heparinized capillary tube and transportation to the laboratory for analysis. Reduced blood pH serves as an early warning sign of acidosis, which is important for the obstetrician [16]. If the blood sample results are borderline (pH 7.1–7.24), a follow-up sample should be taken 30 min later, while birth should be expedited in cases of pH ≤ 7.20 [17].

The condition that ruptures fetal scalp membranes induces risk to mother and child in terms of tissue reactions, pain, bleeding, and infection. These risks are increased in fetuses with suspected hemophilia or thrombocytopenia [15] and in mothers with HIV, hepatitis B, or C [3]. In addition, the analysis requires a large amount of blood (30–50 uL) and is time consuming (median time taken is 18 min) [19], resulting in diagnostic delay while pH drops at a rate 0.01–0.04 unit/min [20,21]. Prolonged collection time increases the risk of blood clotting and clogging the analyzer [22]. Due to the liquid nature of the blood sample, it is easily contaminated by amniotic fluid, meconium, and other fluids that may enter the blood sample and affect the pH measurements [23].

FBS is currently available in two forms of measurement, which are pH and lactate. Lactate measurements have an advantage in terms of analysis because they require a smaller amount of blood (5 μL) compared to pH analysis (30–50 μL) [22]. FBS has shown to be capable of detecting fetal acidosis in up to 10% of cases where ST events were absent on ECG or CTG [2]. FBS also revealed that only 11% of fetuses with suspicious FHR had acidosis, defined by Saling as a scalp pH < 7.20. These findings suggest that FBS could reduce the number of cesarean sections associated with the use of continuous CTG [3].

#### 2.2.2. Non-Invasive Method

There are a few non-invasive fetal monitoring techniques, which are discussed below.

The fetoscope, invented by David Hillis in 1917, was the first non-invasive method for monitoring fetal development. It is an intermittent auscultation (IA) technique used during labor to monitor the fetal heart rate (FHR). IA is the best way to monitor healthy women with healthy pregnancies at a low risk of complications [24]. However, it was not a reliable indicator of fetal distress other than in an extreme situation of terminal bradycardia [25].

Fetal Pulse Oximetry (PO) is a technique for determining the saturation level of fetal blood oxygen (S${\mathrm{pO}}_{2}$) based on the relative absorbance of multiple light wavelengths by oxyhemoglobin and deoxyhemoglobin, which produces a periodic time domain signal called a photoplethysmogram (PPG). PPG pulse depth at two wavelengths is used to estimate the relative concentration of ${\mathrm{HbO}}_{2}$ in pulsating arterial tissue. Conventional invasive PO is performed using a probe placed on the fetus’ head while it is in the uterus and vaginal canal during labor [26,27]. However, transabdominal PO available today have apply near-infrared light transmitted to the maternal abdomen and fetus in order to measure the oxygen saturation in the fetal blood non-invasively [27].

**Figure 2:**
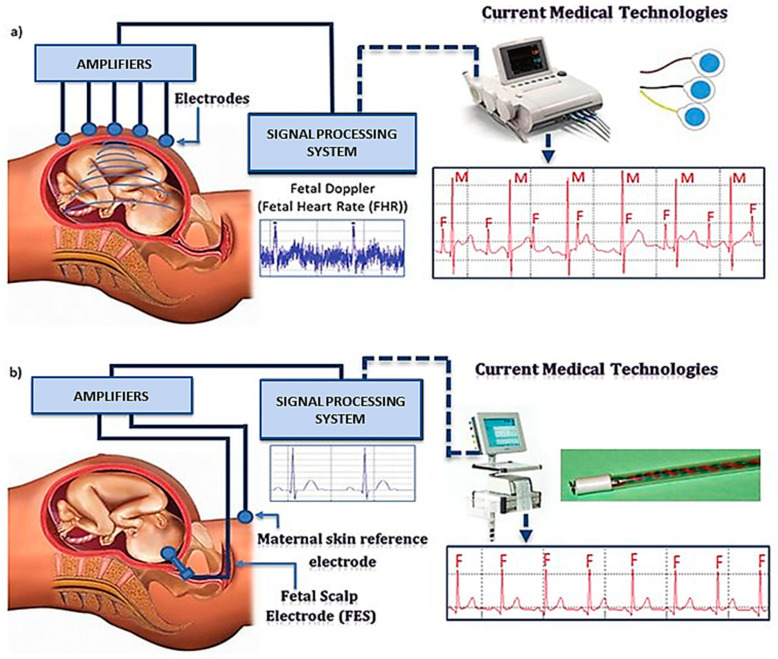
fECG placement and the working principle (**a**) external electrode (**b**) internal electrode. “Adapted with permission from Ref. [28], 2012, Martinek, R.; Žídek, J.”.

The fetal electrocardiograph (fECG) is a graphic record of the electrical activity of myocardial cells that reflects the oxygenation level of the myocardium, as shown in Figure 2. 

**Figure 3:**
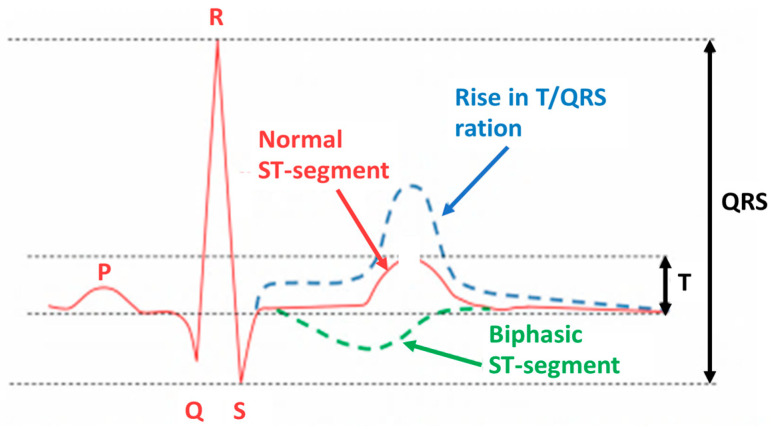
Fetal ECG in acidosis occurrence. “Adapted with permission from Ref. [28], 2012, Martinek, R.; Žídek, J.”.

The fECG classification system incorporates a ST analysis (STAN) algorithm for interpretation. It evaluates changes in the fECG complex (Figure 3), most notably the ST segment and T wave [29,30]. Additionally, the T/QRS ratio is analyzed, which indicates the duration of the hypoxic insult in the cells [29] and correlates to the severity of myocardial damage. ECG bradycardia develops immediately as a result of hypoxia and the resulting acidosis. Bradycardia is a critical time marker for delivery, as any delay in delivery greater than 18 min from uterine rupture results in significant morbidity. The ECG should be used in conjunction with other evaluation tools, such as echocardiography and biochemical markers [5].

Electro myometrial imaging (EMMI) evaluates three-dimensional (3D) uterine electrical activation in detail and visualizes uterine contractions in 3D with high spatial and temporal resolution. EMMI originates from magnetic resonance images of body surface electrical recording combined with body–uterus geometry. EMMI is a technique that utilizes electrodes placed on the abdomen to reconstruct the uterine electrical activation patterns and thus generates CTG. These patterns correspond to those obtained when electrodes are placed directly on the uterine surface [31].

The development of non-invasive methods is an excellent alternative for fetal monitoring, since invasive methods suffer limitations such as bleeding, tissue biocompatibility, and trauma to the fetus and mother. Non-invasive methods could eliminate those adverse effects before irreversible damage to the fetus occurs, thereby lowering mortality and morbidity rates. However, accurate reading is the main constraint. This motivates researchers from several disciplines to develop high precision fetal monitoring devices that employ a variety of techniques.

## 3. Magnetic Induction Spectroscopy (MIS)

The traditional bioelectrical impedance spectroscopy (BIS) technique was executed by a direct contact of electrode with the measuring samples; for example, using an open-ended coaxial probe. Hence, the electrical signal is directly induced into the measured sample. 

MIS introduces a fully non-contact inductive coupling between sensors and sample, alleviating some complications, especially with biological samples [13]. This demonstrates the advantage of MIS in terms of being a non-invasive, non-intrusive, and electrodeless measurement scheme [26], as well as abolishing the difficulties of electrode positioning, electrode-sample interface consistency and intrusive contamination [30]. In addition, MIS eddy current sensors are insensitive to dirt, dust, humidity, oil, or dielectric material in the measuring gap and have been proven reliable in a wide range of temperatures [32]. Furthermore, electromagnetic coupling improves patient safety through the use of contactless procedures [33]. It also provides a very fast response for rapid screening within a relatively short period of time, thus allowing for real-time measurement. 

### 3.1. MIS Theoretical Concept

Magnetic Induction Spectroscopy (MIS) is one of the BIS techniques that uses electrical conductivity of materials as a function of frequency [34]. This technique is concerned with the investigation and measurement of spectra produced when magnetic fields interact with matter or emit electromagnetic radiation.

**Figure 4:**
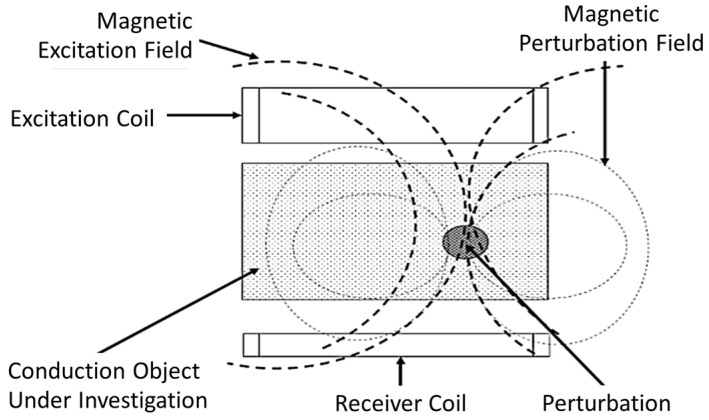
The principle of MIS. “Adapted with permission from Ref. [19], 2016, Sarkawi, S. et al.”.

MIS is a low-cost technique that can be developed simply by using two coils; an excitation coil (Tx) and a receiver coil (Rx) [33], as shown in Figure 4. Tx produces primary fields which penetrate into the tissue. In response to the primary field, an eddy current will flow in the tissue. The tissue will produce its own field known as a secondary field or eddy current field. The signal used to evaluate the material is the change in impedance of the Rx coil. Thus, an alternating current (AC) source must be fed to Tx, which produces a dynamic magnetic field where time-varying magnetic flux around the coil is induced voltage and sensed by Rx [35]. The magnitude of the secondary field that is detected at Rx depends on the conductivity of the tissue and the applied frequency [36]. 

According to Faraday’s law, a time-varying magnetic field induces an electromotive force or voltage in the sample. For a wound coil composed of a number of turns *N*, Faraday’s law is given by:

(6) $$\mathrm{EMF}\;=-N\frac{d\phi }{dt}$$

where EMF is electromotive force, *N* is number of turns of wire, dϕ is magnetic field [37]. 

Lenz’s law states that the direction of induced current is opposite to the current whose magnetic field opposes the change in original magnetic flux. In this case, the secondary magnetic field is directed in the opposite direction to the Tx coil’s external primary magnetic field, resulting in induced current in Rx. Therefore, it produced an opposite sign of induced EMF to the change in magnetic flux ($\frac{d\phi }{dt}$). 

**Figure 5:**
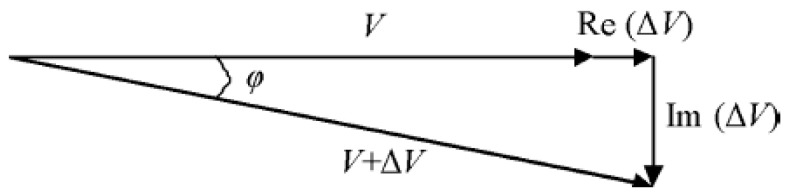
Phasor diagram of Rx coil represents the measured primary signal, *V* secondary signal Δ*V* and the total signal [36].

In the MIS system, the Rx signal is contributed by two components, which are the primary field (*B*) and the secondary field (∆*B*). The sum of *B* and ∆*B* is measured as the associated voltages at the receiver. Therefore, the phasor diagram in Figure 5 is presented to show the detected primary signal, *V* and secondary signal, Δ*V*. Rx signal is a summation of two voltages, *V* + Δ*V*, with lags to the primary voltage by angle φ [6].

Primary voltage *V* is induced by primary field *B* while secondary voltage ∆*V* is induced by secondary field ∆*B*. The relative change in the magnetic field in Rx coil and the corresponding relative voltage are expressed by [38]

(7) $$\frac{∆B}{B}=\frac{∆V}{V}$$

If the Rx coil has received a high voltage, it means that more secondary fields were able to be detected. However, the secondary voltage is usually too small |∆*V*/*V*| ≪ 1, which results in it being overshadowed by the primary voltage. |∆*V*/*V*| can be as low as $10^{-7}$ in the beta dispersion region [39]. This is due to the low conductivity of tissue, usually less than 2 S/m in biomedical measurement. Relative voltage has real and imaginary components related to permittivity, $\varepsilon_{r}$, and conductivity, σ, of the sample, respectively [39,40] as shown in Equation (8).

(8) $$\frac{∆V}{V}\propto \omega \left(\omega \varepsilon_{0}\varepsilon_{r}-j\sigma \right)$$

The interaction of the magnetic field with biological tissues follows Maxwell’s Equations [41] which consists of Faraday’s Law Equation (9), Ampere’s Law Equation (10), Coulomb’s Law Equation (11), and Gauss’ Law Equation (12) as follows:

(9) ∇×E=−jωB

(10) ∇×H=(σ+jωε)E

(11) ∇·D=ρ

(12) ∇·B=0

where *E* is the electric field induced by time-varying magnetic field density, *B* at frequency ω. *H* is the magnetic field produced by the changes in the electric field, *E* over time. *D* is the electric displacement field as a result of free electric charge density and divergence of the magnetic field *B* is always zero, or free of magnetic charges. 

The measurement of biological tissue properties by using magnetic induction is based on a spectroscopy technique. Spectroscopy is a measurement technique used to obtain the complex impedance spectrum of a biological sample that changes with respect to frequency. The electromagnetic spectrum shows the entire distribution of electromagnetic radiation according to frequency ranges or wavelengths. The higher the frequency, the shorter the wavelength, and the greater the energy and ionization. For MIS applications, Tx frequency is usually set in the radio-wave frequency (RF) region (3 kHz–3 GHz). RF is non-ionizing radiation that has insufficient energy to break chemical bonds or remove electrons (ionization). 

The dielectric spectrum of tissue consists of a small part of the RF frequency range. It contains a beta dispersion region, which is the most useful frequency for medical applications. This frequency provides a low specific absorption rate (SAR), thus making it safe for the subject. SAR describes the energy absorbed by tissues as exposed to a magnetic field in power per mass (W/kg). SAR tests are performed for 6 min of exposure. SAR is defined as

(13) $$\mathrm{SAR}\;=\frac{\sigma }{2\rho }E^{2}$$

where *σ* is the conductivity of body tissue (S/m), ρ is the density of body tissue (kg/m^3^), *E* is the RMS value of the electric field strength in the tissue (V/m). Absorption is a function of tissue permittivity, conductivity, and frequency. A weak RF field could induce a current (eddy current) in the tissue. The stronger the field, the larger the eddy current, and thus the higher the induced voltage at Rx. 

Radiation may cause heating. However, a weak RF field is insufficient to cause thermal heating as an effect of vibrating molecules in the tissue. Tissue heating is dependent on source frequency, tissue thickness, dielectric constant, and water content. As the tissue conductivity increases, the energy absorbed becomes higher and heat is generated. Nevertheless, in order to heat the tissue, it requires a relatively large amount of radiation. By using the correct frequency range, it will minimize the effect on the tissue and maximise the system functions.

### 3.2. Dielectric Spectrum of Biological Tissue

**Figure 6:**
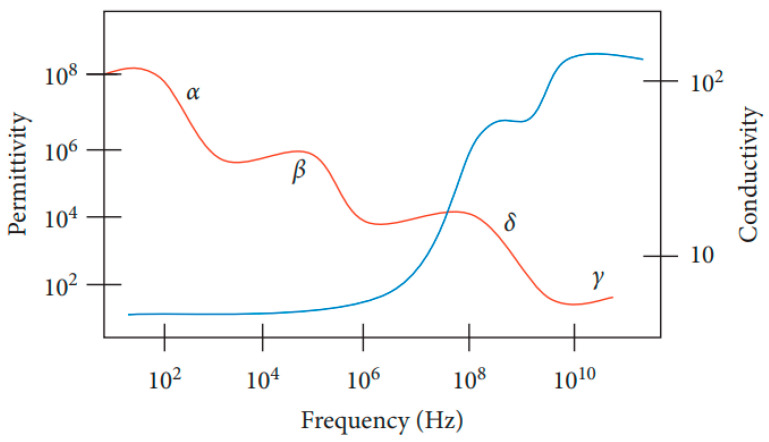
Dielectric spectrum of tissue [42].

Biological tissue is one of the diamagnetic materials with a weak magnetic susceptibility ($\chi_{m}$) around $-10\times 10^{-6}$ [38] dominated by the presence of water. As biological tissue is exposed to a magnetic field, it will cause interaction of the magnetic field with the tissue, which can be explained by the dielectric spectrum of biological tissue shown in Figure 6. This spectrum provides insights into a sample’s properties and its interaction with the electromagnetic field at cellular and molecular levels over a range of frequencies (permittivity and conductivity). 

The four main dispersion regions are alpha α (10 Hz to 10 kHz), beta β (10 kHz to 10 MHz), delta δ (0.1 to 5 GHz) and gamma γ (above 0.1 GHz) with their respective frequency ranges at low, medium, and high frequency (δ and γ) [42]. The dispersion region depends on the nature of tissues and the extent of their ionic content and ionic mobility. Alpha dispersion is associated with ionic diffusion along the cell membranes. Beta dispersion occurs due to interfacial polarization of cellular membranes, proteins, and other organic molecules. Gamma dispersion is due to polarization of water [43].

**Figure 7:**
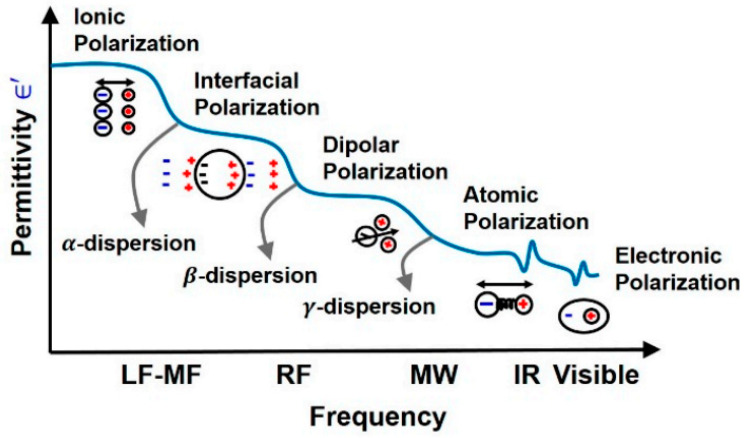

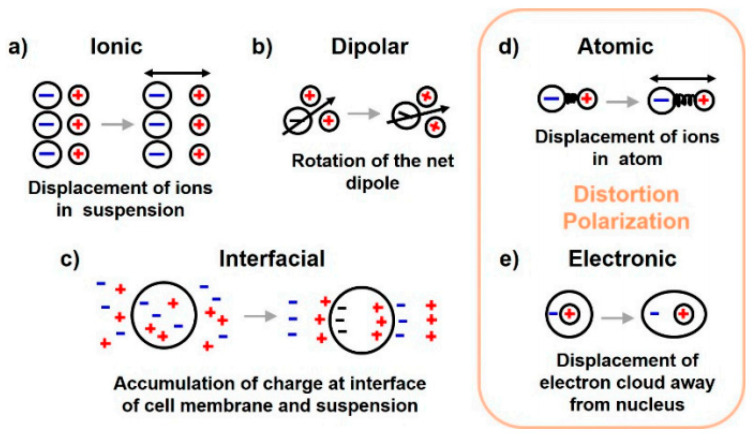
Polarization mechanisms of dielectric materials across different frequency ranges [44].

Dispersion regions are divided according to the types of polarization shown in Figure 7. The polarization effect increases with increasing sample conductivity [43]. The order of polarization and relaxation mechanisms are observed when an alternating electric field of increasing frequency is applied to biological matter. Ionic diffusion (a) is observed first, followed by (b) interfacial, (c) dipolar relaxations, (d) atomic, and (e) electronic resonances [44]. The beta dispersion region occurs at the frequency range where dipolar polarization occurs, causing rotation of the net dipole. 

Permittivity is the ability to store charge or rotate molecular dipoles and is also the speed of light travel in a material [43] explained by polarization. Debye parallel RC element explains the magnetic field penetration in biological tissue. The process starts with membrane reactance short-circuiting the membrane resistance, which finally allows an electric field to penetrate into the cell interior. The Debye expression approximates the complex relative permittivity ($\hat{\varepsilon }$) as a function of angular frequency (ω) at a single time constant (τ) given by

(14) $$\hat{\varepsilon }=\varepsilon_{\infty }+\left(\frac{\varepsilon_{s}-\varepsilon_{\infty }}{1+j\omega \tau }\right)\;\;$$

where $\varepsilon_{\infty }$ is permittivity at field frequency where ωτ≫1, and $\varepsilon_{s}$ is permittivity at ωτ≪1  and $j^{2}$ = − 1. Permittivity develops the electrical field in materials.

**Figure 8:**
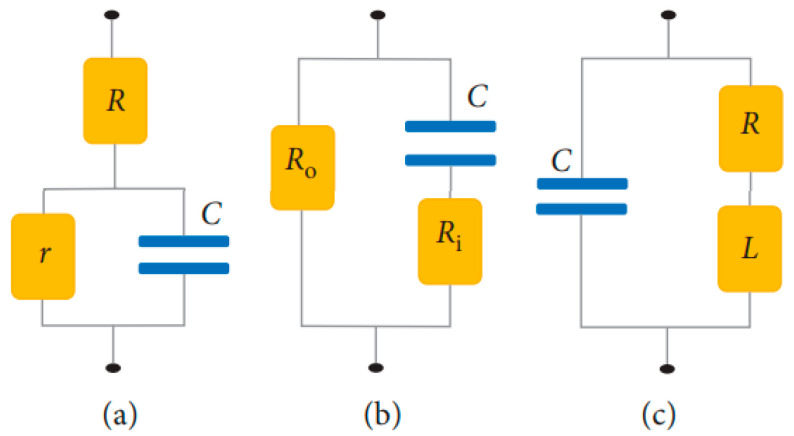
Designs of equivalent RBC circuit by (**a**) Philippson; (**b**) Fricke and Morse, and (**c**) Cole and Baker [42].

The variation of permittivity with frequency can be explained by using an electrical circuit model in terms of capacitance and conductivity. Scientists have proposed various equivalent RBC circuits [42]. Figure 8a shows Philippson’s circuit where *R* is the resistance of cytoplasm, *r* is the membrane resistance, and *C* is the membrane capacitance [45]. Fricke and Morse proposed a RBC suspension circuit (Figure 8b), where $R_{o}$ is resistance around the cell and $R_{i}$ is the resistance of cytoplasm. Cole and Baker worked out an inductive reactance within the membrane structure in Figure 8c. Components of R, L, and C have values of 1 ${k\Omega \mathrm{cm}}^{2}$, 1 $\mu F/{\mathrm{cm}}^{2}$, and 0.2 ${\mathrm{Hcm}}^{2}$, respectively. Cole–Cole dispersion is used to represent the biological tissue as an electronic circuit by a parallel combination of a resistor and a constant phase element (CPE). This accounts for the frequency dependence of each tissue region.

Cell resistance and capacitance are quantified by the parameter (ϕ). ϕ signifies the ratio of actual cell membrane area to the membrane area ($4\pi r^{2}$) formed by smooth and spherical layers of cytoplasm or nucleoplasm [42]. An approximate value for the effective capacitance, *C* of Philippson’s proposed equivalent circuit of a membrane can be found using

(15) $$C=\frac{\varepsilon_{0}\varepsilon_{r}\;A}{d}$$

where $\varepsilon_{0}$ is the permittivity of air,$\;\varepsilon_{r}$ is permittivity of materials, *A* is the area, and *d* is the thickness of the membrane. The lower the resistance, the higher the magnetic permeability. 

**Figure 9:**
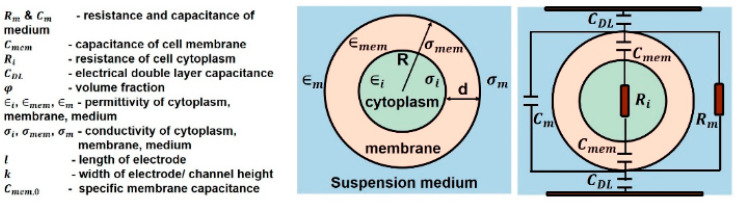
Equivalent circuit model of a single cell in suspension medium [44].

The impedance of a single cell in suspension can be determined by using two parallel electrodes. Figure 9 shows an equivalent circuit model for a single cell in suspension medium. The impedance value of a single cell is derived by using Maxwell’s mixture theory. The impedance of the medium is represented by a parallel resistor $R_{m}$ and a capacitor $C_{m}$. Cell membrane $C_{mem}$ has a very low conductivity, which prevents current flow through the cell at low frequency (<1 kHz). Therefore, the sensitivity of detection decreases as the majority of voltage is dropped across the cell membrane. Hence, this frequency range is rarely used because it cannot provide information about the cell’s properties. Cell cytoplasm is represented by resistor $R_{i}$ and electrode–electrolyte interface is modeled as capacitor $C_{DL}$ known as electrical double layer $E_{DL}$. The cell membrane has an approximate capacitance of 1 μF/cm^2^ [44].

Conductivity is the capacity of a material to transport charge throughout its volume when an applied field is applied, or the capacity of materials to allow current to flow [43]. Conductivity occurs as a result of ionic drift and mechanisms of lower-frequency polarization. It is limited by the scattering of electrons caused by irregularities in the structure’s periodicity, such as vibrations and impurities.

**Figure 10:**
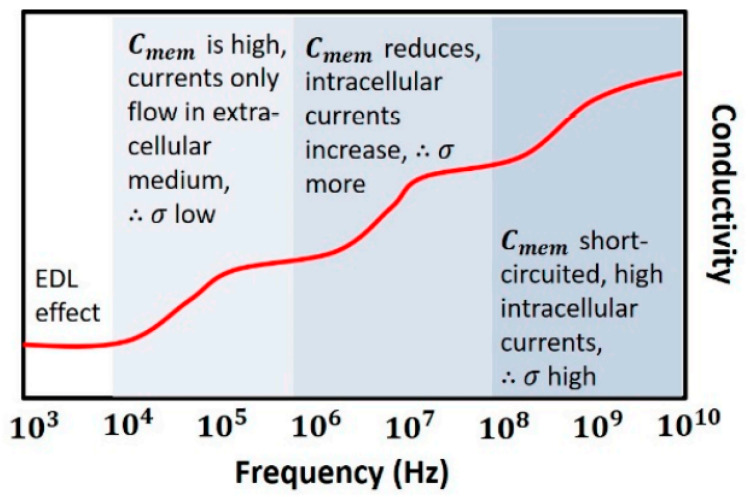
Conductivity of dielectric with increasing frequency of electric field [44].

As shown in Figure 10, at low frequencies (<1 MHz), $C_{mem}$ acts as an insulating layer (low conductivity), thus currents flow in the extracellular medium only. The intracellular information is not accessible since currents cannot pass through the cell. When frequency is increased (1–100 MHz), the cell’s capacitive reactance $C_{mem}$ reduces, the eddy current increases, and consequently, conductivity is increased. As the frequency is increased further (>100 MHz), $C_{mem}$ is effectively short-circuited, thus enabling external fields to pass through the cytoplasm. The signal becomes more sensitive to the change in intracellular content and reaches its maximum conductivity [44].

Thus, it is evident that at low frequencies of (α and β-dispersion region), the information associated with the cell membrane and extracellular fluid (cell shape, size, and membrane potential) can be obtained. These low frequencies have been applied for characterization of different white blood cells (WBC) including monocytes, lymphocytes, and neutrophils, based on their independent shape and morphology, thus acquiring different $C_{mem}$. for classification purposes. This distinction is made on the basis of cell membrane potential by subjecting cells to a frequency within the kHz–MHz range [44]. It is best explained by the properties of live cells, which contain a large number of negative charge molecules that attract positive charges (sodium and potassium ions) from the suspension medium.

Conductivity is affected by the magnetic permeability of a cell. The magnetic permeability of a material is the ability of a material to support the formation of a magnetic field inside itself, or a measure of the magnetization of a material [43]. Permeability of non-magnetic materials such as biological tissue is considered ($\mu_{r}$ = 1) [39,46]. Magnetic permeability is a scalar in isotropic mediums. Free space has a characteristic permeability constant *μ*_0_. Permeability of materials is expressed as relative permeability $\mu_{r}$ with respect to free space $\mu_{0}\;$ as in the equation below:


(16) $$\mu =\mu_{r}\mu_{0}$$

### 3.3. MIS in Various Application

MIS is well-known in industry for its use in search coils and non-destructive testing of cracks and flaws in metal [36]. It is capable of classifying non-ferrous metals (copper, aluminum, and brass) [47]. Furthermore, it is capable of analyzing the inductance curve, distinguishing discrete microstructural states, and determining the heating and cooling process’ phase transformation points [48]. MIS has previously been successfully used to determine the pH of water for water and wastewater treatment, with changes in hydrogen bonding cited as the source of observed pH effects [49]. In addition, it was designed on a food industrial scale to test samples of yeast suspension, apples, oranges, and tomatoes. MIS provides a method for measuring bioimpedance spectroscopy, which is conductivity without any contact with the test sample for various concentrations [50].

MIS also has great potential in biomedical engineering applications, especially due to its non-contact measurement capability [34]. MIS has been used on rabbits as a brain diagnostic device to differentiate between two types of strokes which are hemorrhagic and ischemic [51]. MIS demonstrates a strong ability to detect screw cracks in implants [52]. Additionally, it was suggested as a method for assessing breast cancer [53] where it was noticed that tumors have a higher conductivity relative to normal surrounding tissue [43]. MIS also performs well after being tested as a probe for cervical tissue measurement [54].

### 3.4. Scalp Tissue Characteristics

**Figure 11:**
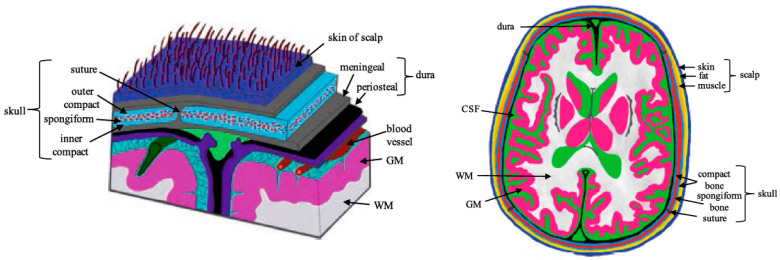
Cross-section of the head with underlying tissues and the detailed layers of the scalp, skull and brain. “Adapted with permission from Ref. [55], 2019, McCann, H.; Pisano, G.; Beltrachini, L.”.

Scalp is a type of skin that contains various types of cells which carry out different functions individually. Scalp consists of several layers, namely skin, blood, fat, muscle, and skull, as shown in Figure 11. Scalp exhibits a variety of intriguing properties as a result of its highly inhomogeneous structure, which results in inhomogeneous dielectric properties.

Skin is generally a laminar tissue composed of three layers which are the epidermis, dermis, and subcutaneous tissue. The epidermis is the outer layer of skin, with a thickness ranging from 0.05 to 1.5 mm on the eyelids and palm. The epidermis is composed of numerous layers. The outermost thin layer, stratum corneum (SC), is around 20 mm, where it contributes the most dielectric properties of skin [45]. That layer is composed of dead skin cells that shed every two weeks. It is significantly less hydrated than the deeper granular tissue. The dielectric properties of composite skin would fall within the bounds formed by the two components [56]. Its high resistivity makes skin one of the most resistive tissues in the human body, which provides the protective barrier between the body tissues and the environment [45]. The rest of the epidermis layer is necessary for the immune response. The dermis layer provides firmness and elasticity to the skin. Subcutaneous tissue, which consists of fat, connective tissues, nerves, and blood vessels, exhibits lower resistivity [43].

Measurement of the dielectric properties of a tissue is not straightforward due to several factors, including tissue inhomogeneity, anisotropy, and its physiological states. Tissue homogeneity is related to the structure due to the multiple cell sizes and functions carried out by the cell. All tissues are isotropic except muscle and bone (anisotropic). Anisotropy is the property of being dependent on the direction (longitudinal, transverse, or three orthogonal planes). Thus, data for the transverse and longitudinal directions are usually presented separately [43]. However, anisotropy of muscle measured at three mutually perpendicular directions minimally affects the electrical conductivity by ±0.02. This is due to a lack of rigorous correspondence between the coaxial probe geometry and tissue structure [57]. Electrical properties are also affected by temperature, where the mobility of ions increases due to decreasing fluid viscosity. It is thus difficult to extrapolate from the dielectric properties of a cell suspension to those of an intact tissue [43]. 

For the development of the MIS pH probe, the sample is assumed to be a homogeneous, linear, isotropic, resistive, and non-magnetic medium [58].

Scalp thickness is described by the variation of components in the geometrical head model. The thicknesses of six major tissue layers are compared as shown in Table 2. The incorporation of different scalp tissues is crucial for safety and wireless system performance. This also gives information to define the minimum and maximum distance between the scalp and the MIS probe. In simulation, the tissues should be assigned to their corresponding dielectric properties as described in Section 3.5.

### 3.5. Dielectric Properties of Scalp

For the application of MIS as a non-invasive method, the presence of several scalp layers—namely skin, blood, fat, muscle, and skull—need to be included (Figure 11). This is because each layer has a different conductivity and permeability measurement. The high impedance of the skin may contribute to measurement errors [54]. The pH measurement will ensure that it is minimally affected by including the existence of different types of tissues. However, it is known that all biological tissue is not a perfect dielectric and its pH is time- and space-dependent [43].

**Figure 12:**
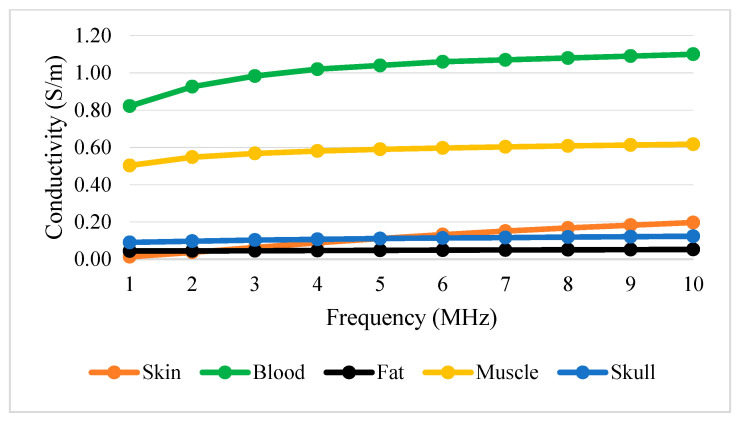
Tissue conductivity versus frequency.

**Figure 13:**
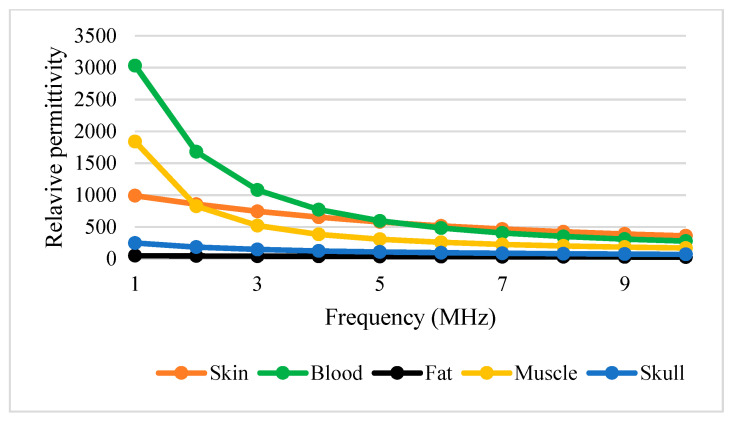
Tissue permittivity versus frequency.

Table 3 shows conductivity and permittivity for different tissues ranging from 1 to 10 MHz [63,64]. Figure 12 and Figure 13 simplify the data presented in Table 3, thus providing a clear view of the variation of conductivity and permittivity for each tissue. Blood shows the highest value for both conductivity and permittivity at frequencies below 5 MHz. It is followed by muscle, skull, skin, and fat [43]. The lowest conductivity value was recorded by skin at 1 MHz with $1.32\times 10^{-2}$. S/m and the highest was blood at 10 MHz with 1.10 S/m. The lowest permittivity value was recorded by fat at 10 MHz with $5.26\times 10^{-2}$ S/m and the highest was blood at 1 MHz with 3.03 × 10^3^ S/m. Fat conducts electricity poorly compared to water. Thus, changes in the percentage of body fat or water are reflected in tissue impedance changes [56]. These data are supported by the previous study that mentioned that conductivity is dominant [65], affected by the presence of 90% water. 

Figure 12 and Figure 13 show that permittivity decreases and conductivity increases when frequency increases, as indicated by the bioimpedance spectrum. At low frequencies, biological tissue will act as an insulator because it contains dipoles and charges that move in a restricted manner. Increasing frequency will increase the conductivity due to the increasing displacement current. This can be described by active and passive ions and molecules transported through a selectively permeable membrane. At higher frequencies, capacitive effects are more important and tissue will behave like a conductor [43], as shown by the increasing conductivity of all tissues in Figure 12.

Study [43] shows that the high impedance of skin is dominated by the stratum corneum layer, although it is very thin. At frequencies less than 10 kHz, 50% of total impedance is contributed by skin, but it drops to 10% at 100 kHz. In addition, the anisotropy property is also more pronounced in the low-frequency range (<10 kHz). These differences are greater in the conductivity than in the permittivity data. However, if the current is applied at a high enough frequency (MHz range), the muscle anisotropic properties will disappear and the conductivity will increase with time [43]. 

There is limited literature indicating the dielectric properties of fetal tissues. However, it was observed that the permittivity and conductivity of adults varied from those of 7-year-old children, as shown in Table 4. Thus, the fetus is also expected to have higher values of conductivity and permittivity compared to 7-year-old children. These data are crucial for the accurate determination of pH by using passive electrical properties, which are dominated by conductivity [65]. 

## 4. Non-Invasive MIS Probe Design Specifications for Acidosis Detection

Coils have been used by MIS researchers whereby the magnetic field measurement component was based on electromagnetic induction theory. A basic MIS setup was developed by using a copper coil assembly connected to a source signal in sinusoidal form and a voltage measurement device. The effect of source frequency alteration on the secondary coil voltage was studied for different coil turns, coil diameters, and coil materials.

The coil wire, shape, and dimension were chosen specifically to perform a particular function. There are various coil types presented in magnetic induction applications based on the arrangement of Tx–Rx. The most common types are planar coils, gradiometers, and perpendicular coils. The intended signal for MIS application is the secondary field. However, the primary magnetic field is known to be higher than the secondary magnetic field, which is up to 100 times weaker (0.1% of primary magnetic field). The coil positioning of Tx and Rx was adjusted to reduce the effect of the primary signal in the resulting signal as it overshadowed the intended secondary signal. Optimization of the coil sensor can cancel the primary field by applying a few designs such as planar coil, gradiometer, and perpendicular coil.

The following subsections will discuss MIS instrumentation. It describes the coil design specification. It includes the type of coil structure, coil materials and coil core, coil turns and diameter, skin effect, lift-off, excitation current, and frequency. It also includes the working principle of each coil type in detail. 

### 4.1. Types of Coil Structure

**Figure 14:**
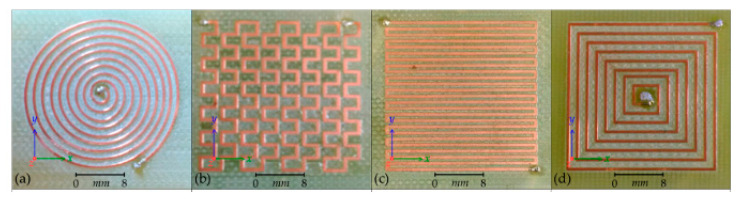
Types of planar coils. (**a**) Circular coil; (**b**) mesh coil; (**c**) meander coil; (**d**) square coil [67].

The first and most basic type of coil is the planar coil. Planar coils are generally flat spirals of conductive track mounted on a flexible polymer or PCB (printed circuit board) substrate. The shape, number of turns, width, and thickness of the track can be varied. Other classes of planar coils are the meander and mesh coils, which do not spiral but alternate directions across the substrate with varying track thickness and displacement, as shown in Figure 14. 

**Figure 15:**
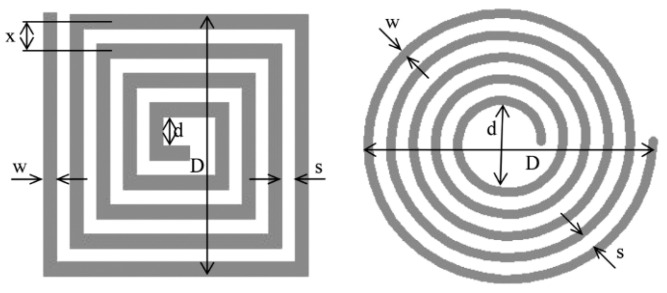
Dimensions of planar square coil and circular coil [69].

Spiral coils describe the eddy current circumference in the sample and offer attractive features of sensitivity [32]. However, in crack detection, a rectangular coil shows a larger detection signal compared to a circular one since a rectangular detector coil has a larger interaction zone, thus obtaining a lower signal-to-noise ratio (SNR) and also reducing the lift-off noise [68]. A previous simulation study of pH shows a comparison of different MIS probe setups, including linear, circular, and square coil designs. A circular coil was chosen to be the most suitable coil for the MIS design after being fed with 1A of current at Tx. It has recorded very good sensitivity compared to linear and square coils in the detection of fetal acidosis. This is due to the high amounts of magnetic field and current density that have been received at the Rx coil [65]. The variation in square and circular coil parameters is shown in Figure 15.

**Figure 16:**
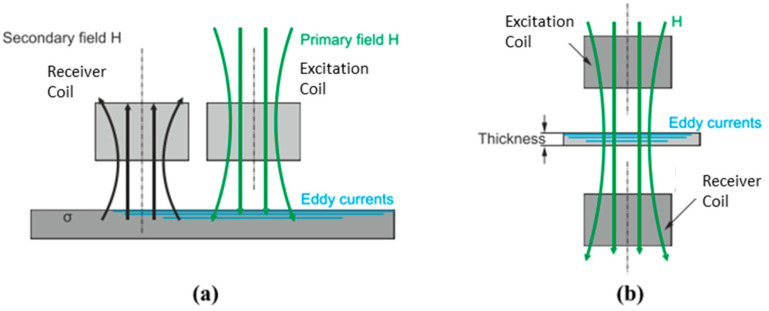
Planar coil of Tx-Rx arrangement for (**a**) conventional and (**b**) transmission method [32].

Common types of coil arrangement for planar coils are conventional and transmission methods. The most widely used is the conventional method. It consists of positioning the Tx and Rx side by side in front of the inspected material as shown in Figure 16a. It is also known as a coplanar arrangement. On the other hand, the transmission method is used for separate-function probes where Tx is coaxial and concentric to Rx, as illustrated in Figure 16b. The transmission method requires a maximum thickness of testing material up to 3–5 times the standard penetration depth in order to receive the signal at Rx [32].

**Figure 17:**
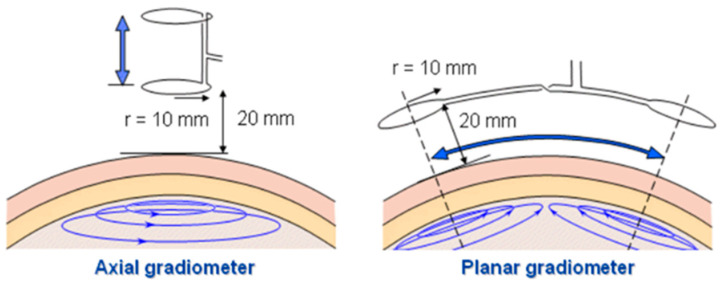
Measurement configuration of axial and planar gradiometer. “Adapted with permission from Ref. [71], 2004, Malmivuo, J.”.

The second type of coil is the gradiometer. A gradiometer is a coil that operates in an antiphase connection of coils, canceling induced voltage from the primary magnetic field while maintaining desired voltage from the secondary magnetic field [54]. The principle is based on the fact that, in a uniform field, two identical and perfectly aligned sensors will give identical outputs, which can be subtracted from one another to give a zero output, effectively eliminating the apparent presence of the field. Gradiometer coils can be divided into a few types, namely planar, axial, and asymmetrical gradiometers [36,70], as shown in Figure 17. This paper only presents the basic gradiometer, thus excluding discussions of the asymmetrical type.

Figure 17 shows planar gradiometers of 10 mm coil radius at a 20 mm recording distance for magnetoencephalography (MEG) lead system [70]. An axial gradiometer detects the largest signal a couple of centimeters away from the site of the local source (arrow), whereas the planar gradiometer detects the maximum signal just above the source. The signal in the planar gradiometer depends strongly on its orientation. If it is rotated by 90°, the obtained signal would in this case vanish [72]. A significant sensitivity enhancement was demonstrated by using a gradiometer coil compared to a single-sensing coil. In a fluid conductivity study [39], the gradiometer sensitivity was measured to be −1.91°/(Sm^−1^). Residual voltage, *Vres* = *V*1 − *V*2 is produced by the gradiometer arrangement due to the non-ideal symmetry of the gradiometer coil [39]. The gradiometer technique could improve the ability to reject common mode component by increasing its order from first-order, second-order (combining two first-order gradiometers), and third-order [35]. 

Planar gradiometer (PGRAD) is capable of canceling far RF interferences to a greater extent through differential design [36]. The advance of the gradiometer is called a zero-flow gradiometer (ZFGRAD) in the form of perpendicular orientation positioning with respect to the Tx coil. This configuration has produced a zero net primary magnetic flow in it. The immunity of ZFGRAD to far magnetic field perturbation improved 2–12 times compared to PGRAD and zero flow coil (ZFC). 

**Figure 18:**
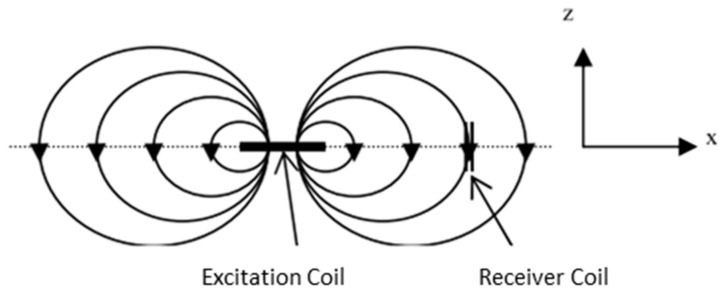
Perpendicular coil arrangement of Tx-Rx coil [36].

The third type of coil is perpendicular. Perpendicular coils show the arrangement of Tx, which is perpendicular to Rx. Inductive sensors are sensitive only to the flux that is perpendicular to their main axis [39]. Perpendicular coils work by compensating the signal through the perpendicular arrangement of Tx towards Rx as shown in Figure 18. 

As the Tx coil is perpendicular to the sample, the change in magnetic flux is given by the magnetic flux passing through the coil area as follows:

(17) ϕ=BA cos θ 

where ϕ  = magnetic flux, *B* is magnetic field strength, *A* is the area of the coil perpendicular to the magnetic field, and θ is the angle between the magnetic field lines and the state of the coil [73]. A maximum magnetic field strength will be detected as the coil is at 0° to the magnetic flux, *ϕ* and reduces its strength as it approaches 90°. By locating Rx coils parallel to magnetic field lines, magnetic field cancelation of eddy current can be achieved theoretically. 

**Figure 19:**
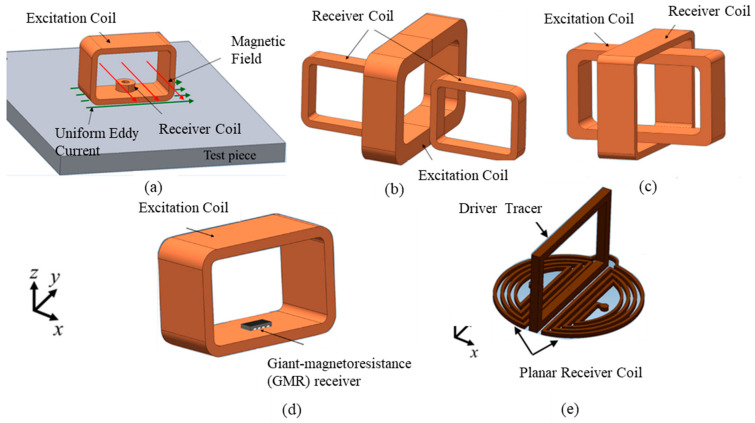
Different types of perpendicular coil. (**a**) One-Direction Hoshi (ODH) probe (**b**) Plus-Probe (**c**) Cross probe (**d**) Probe with a Giant-Magnetoresistance (GMR) Detector (**e**) IOnic Probe [68,75].

Another design shows a model of perpendicular coil where Rx is placed above the center of Tx [73,74] so that the primary magnetic field is always parallel to the coil plane (*θ* = 90°) and magnetic flux, ϕ=0. This will generate zero induced voltage because there will be no change in magnetic flux over time. So, the primary magnetic field is eliminated [74]. This is defined as the “perfectly aligned” position of the proposed system [73]. There are other configurations of perpendicular Tx-Rx as shown in Figure 19a–e. 

A three mutually perpendicular coil design is considered to be used for the purpose of determining all directional components of a magnetic vector—three-axis magnetic field measurements [35]. This is in view of facts that the Rx coil is sensitive to the flux that is perpendicular to the main axis only.

### 4.2. Coil Materials and Coil Core

Coil materials are made up of electrical conductors, usually copper or other non-ferromagnetic materials, to avoid magnetic hysteresis effects. Copper has a great advantage in terms of low resistivity, high conductivity (58.4 MS/m) and low-cost material. Thus, it is very good for inductive sensor applications. A fill factor is a ratio between the area of the electrical conductors and the size of the winding space. When wires are packed tightly together, the quantity of air space is reduced and the fill factor is increased. Increasing the fill factor can be achieved by using rectangular, square, or flat coils. This, in turn, improves the efficiency and conductivity of an electrical device.

An electrical coil is made up of a core and wire that wraps around it. Some electrical coils are built entirely of wrapped wires and do not contain any core. The type of material used to make the core determines the magnetic field strength and coil inductance, and thus the overall performance of the coil. The coil core acts as a magnetic flux concentrator [35]. The two types of core that are most commonly used are air-cored and ferrite-cored. 

Air-cored coils are unaffected by the current they carry, since they have no core for their inductance. They have an advantage as frequency increases as unlike ferromagnetic coils, they are not affected by iron losses. An air-cored coil may operate at up to 1 GHz compared to a magnetic-cored coil. The air-cored system induced a higher current density in the surrounding saline than in the saline near to the probe face. Since no magnetic concentrator was used, the signal falls off less rapidly. A fitted power curve ($y=ax^{-2}+b$.) indicates that the induced current density falls off at a rate $1/r^{2}$ [32]. 

Ferrites have a greater permeability than air-cored coils, thus the initial coil impedance is higher. For the ferrite-cored system, the induced current density is distributed near the probe face similar to the coil radius and is confined by the probe geometry. However, when z > 30 mm beyond the surface of the cervix tissue sample, the ferrite-cored probe shows that Jphi falls off rapidly and reduces to <5% [32]. The disadvantage of the ferrite-cored type is it that it introduces non-linear factors where the permeability of the coil transfer function depends on the magnetic field value, temperature, time, and frequency. Thus, it has poor stability. Nevertheless, if the core is well-designed, these effects can be significantly reduced [35,74].

Thus, air-cored coils are used due to their high stability, despite their lower sensitivity [74]. The most important advantage of an air core is its linearity. In addition, the air-cored probe is more sensitive to deeper tissues than the ferrite-cored probe. Thus, air cored is preferable. The flux density *B*, for a non-ferromagnetic medium is given by *B = µ·H* where (${µ}_{0}=4\pi \times 10^{-7}{\;\mathrm{Hm}}^{-1}$) and the sensitivity of an air-cored coil sensor is defined as the slope of the output system [35].

### 4.3. Coil Turns and Diameter

The number of coil turns could be fixed to a certain range to improve and optimize the sensor sensitivity. A study observed that the secondary voltage differs for frequencies of 25 kHz–1 MHz with 5, 10, and 20 turn ratios. The secondary voltage was the lowest for five turns and increased as the number of turns increased. It can be concluded that when the number of turns increases, the rate of change in secondary voltage over frequency also increases [33]. For fetal acidosis detection, higher sensitivity was achieved using 5 turns and 12 turns for Tx and Rx, respectively, compared to five turns for Tx and eight turns for Rx coils [65].

Coil size, which is determined by the diameter, should also be put as a top priority to obtain high-level signal detection. At the same time, it is crucial to ensure that the fill-factor is close to one in the case of encircling coil probes [32]. High-sensitivity probes lead to a large number of turns, resulting in a large probe area. An increase in the number of turns will result in a bigger coil diameter, especially for a planar coil. A bigger Tx coil can produce a large current, thus producing a big enough field for detecting conveniently. However, this becomes a conflict for the wide coil turns and resolution requirements, which need to be fit into a limited space of a small probe dimension [37] for the compactness of the whole device. 

### 4.4. Skin Effect

**Figure 20:**
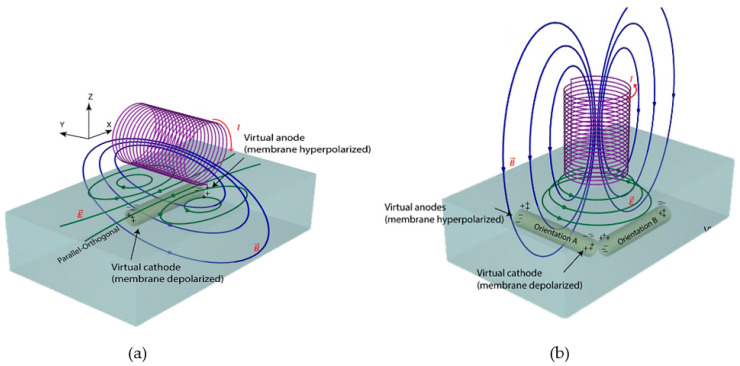
Direction of eddy current flow indicates by green line; (**a**) unidirectional eddy current flow (**b**) circular form eddy current flow [76].

The figure below shows the generation of eddy current (EC) in a sample with its associated direction. The direction of an eddy current depends on the direction of the primary coil current due to the Lenz law. Figure 20a shows a unidirectional EC, while Figure 20b shows a circular EC.

**Figure 21:**
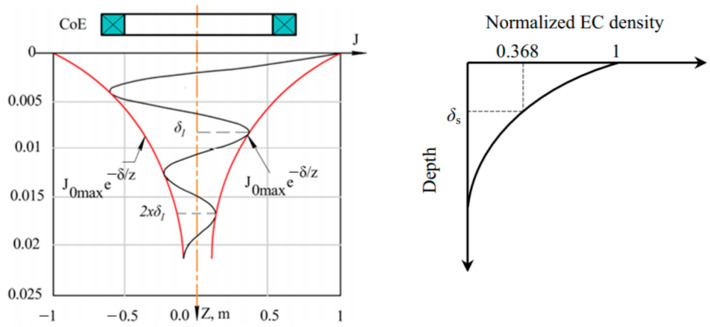
Skin depth shows the distribution of eddy current density on the surface of object under investigation [75,77].

The EC flow is not evenly distributed throughout the entire sample volume. The skin effect explains this condition where the EC flow density is maximum just on the conductor surface and exponentially decreases along the depth direction. Figure 21 shows the electromagnetic field skin depth, which essentially determines the depth of current flow. The electromagnetic field skin depth at frequency f for an AC is given by

(18) $$\delta =\frac{1}{\sqrt{\pi f\sigma \mu_{0}\mu_{r}}}$$

where σ is the electrical conductivity and $\mu_{0}$ and $\mu_{r}$ are the permeability of free space and relative permeability of the object [46,77]. 

The phasor of EC density along depth (*z*-axis) is given by

(19) $$J_{x}\left(z\right)=J_{0max}\;e^{-\frac{z}{\delta }}\;e^{j\left(\alpha_{0}-\frac{z}{\delta }\right)}\;$$

where $J_{0max}$ is the maximum current density at the surface, and *z* is the depth. The standard penetration depth δ. is the depth at which the eddy current density decreases to a level of about 37% of its initial value at the surface [32], and the magnetic field strength is 1/e where e ≈ 2.7183 [78].

The thickness of the sample must be two or three times the standard penetration depth to avoid EC flow from appearing on the other side of the sample [32]. If the skin depth of the electromagnetic field in the sample is large compared to the thickness of the sample, the ratio between *B* and ∆*B* is proportional to the conductivity of the sample and the frequency of the system [33]. When skin depth δ is usually found to be large compared with the thickness of the target object, the skin depth can be ignored [46].

(20) $$\frac{∆B}{B}\propto \;\omega \sigma$$

The conventional MIS system was useful for the detection of metal cracks at the surface and near-surface up to several millimeters in depth. One way to enhance the subsurface testing is by reducing the operational frequency so that the standard skin depth is increased. However, considering Faraday’s voltage law states that the induced voltage in coil sensors is proportional to the rate of change in the magnetic field, the signal-to-noise ratio is decreased in many instances. To compensate for their high permeability and penetration into the sample, low-frequency tests are normally employed in the inspection of ferromagnetic materials [32].

### 4.5. Lift-Off

Lift-off is the impedance change that happens as the distance between the inspection coil probe and the sample changes over time. Lift-off is stronger as it gets closer to the probe because the magnetic field is stronger near the coil. The lift-off variations are caused by irregular sample surfaces, varying tissue thicknesses, or the operator’s movements. It is considered a noise source. Therefore, the distance between the probe and sample must be as constant as possible in order to avoid lift-off. Reduction of the lift-off effect can be achieved by optimizing the coil design of Tx and Rx. The lift-off and inclination of a pancake-type coil is extremely sensitive with respect to a flat surface sample. In addition, it can scan smaller areas compared to encircling coils [32].

Measurements for ferrite-cored probes were conducted by increasing the distance between sample and probe from 2.5–12.0 mm [54]. Studies show comparison of gap from 3–9 mm between coil and sample where increasing the gap or lit off will reduce the induced voltage thus affect the sensitivity [74]. A phantom-based biomedical application was able to detect a conducting tube with an average conductivity of 0.2 S/m at a distance of 6 cm from the magnetic sensor [79]. The system claimed to have a frame rate of 10 frames/s, hence enabling real-time data processing and also online data updates [79].

### 4.6. Excitation Current and Frequency

In a practical setting, the Tx current is limited by safety regulations to the maximum allowed specific absorption rate produced by the EC. As it increases quadratically with the frequency, the maximum allowable current must decrease with the square of the frequency. The current must be set so that a 10 nV signal can be detected in order to resolve the frequency changes at least 10 mS/m, which is the reasonable upper limit for biological tissues [38].

Tx frequency can be in single or multiple forms. Single-channel frequency excitation measures only a single frequency, so the selectivity is significantly diminished. Measurement of biological samples at a single frequency of a time-varying magnetic field is a challenge as there is a lack of information to be a predictive model. Reconstructing spectral and frequency differences is practical for the measurement of sample conductivity with anisotropic characteristics [79]. This is because a single sinusoidal excitation system is strongly limited by the penetration depth of eddy currents [32]. 

The detectability of MIS can be enhanced by using multi-frequency excitations. This is based on the fundamental principle of conductivity, which is frequency dependent [79]. The output signal at Rx will increase almost linearly with frequency up to the resonance frequency,$\;f_{r}$. Above $f_{r}$, the output signal will drop due to the influence of capacitance [35]. $f_{r}$ is depends on the coil inductance *L* and capacitance *C* given by

(21) $$f_{r}=\frac{1}{2\pi \sqrt{LC}}$$

Multi-frequency excitation works by using two or more frequency values with frequency components spread across the bandwidth of interest. Incorporating various frequency components with a 1/f amplitude ratio into the analysis allows for enhancement of the correlation of a more sophisticated regression model. Measuring an extended bandwidth incorporated with a wider frequency range could extract and connect relevant information about the conductive processes to relate to the actual mechanisms and changes in physiology and morphology at a microcellular level. For instance, using the spectroscopy technique, an impedance ratio is applied at different frequencies to divide out some of the cofounding variables [50].

Multiple frequency data can be reconstructed simultaneously to exploit the correlation among conductivity distributions at different frequencies. Different excitation frequencies were utilized in order to obtain greater linearity and sensitivity [32]. Each of the proportioned frequency components has a good compromise between sensitivity at high and low frequencies. [80]. High-frequency measurements provide information regarding the properties adjacent to the sample surface, while low-frequency measurements sense deeper inside the sample [79]. Thus, different excitation frequencies enrich the information, improving the inverse method and strengthening the system against experimental noise [80]. In addition, multi-frequency techniques allow simultaneous measurement, thus expanding the capability of single-frequency testing and saving time. 

Multi-frequency testing is also applied to cancel out external noise signals in order to improve the signal-to-noise ratio. It uses a composite signal and subtracts the undesirable signal. The sources of noise that can be minimized are temperature variation, probe lift-off and geometrical changes in the sample [32]. The two most challenging aspects of multi-frequency implementation are conditioning electronics and software control. Hence, it is preferable to build a Tx coil with a range of excitation frequencies suited for pH detection. This will enhance the amount of data supplied to the MIS system, making the Rx signal more resilient to anomalies [79].

## 5. Future Design of MIS Probe

Labor usually takes less than 8 h with ≥1 cm dilation per hour. However, the total time of labor can last up to 18 h. The MIS pH probe for acidosis detection is designed to be used as early as the latent phase. The latent phase is the first stage where the cervix becomes fully effaced and dilated to 3 cm. In the active phase of labor, the cervix dilates from 4 cm to full dilatation (10 cm) [81]. Fetal scalp blood sampling can only be used during the active phase due to the large amnioscope size [3]. Thus, the MIS probe will become more convenient for early acidosis detection.

### 5.1. Design Considerations

Medical devices should be used in a safe and efficient manner throughout the expected life cycle of the product. Thus, the design of medical devices should implement measures in all aspects, such as correct, timely, and secure data transmission. G. Cummins et al. [1] has given a clear outline of the desirable device requirements for detection of fetal acidosis. Four important parameters for an ideal device are: (1) ability to maintain accuracy and functionality once it is exposed to the external environment; (2) possibility of obtaining biological fluid samples, such as interstitial fluid or blood; (3) appropriate device size and specifications; (4) biocompatibility [1]. 

For MIS acidosis detection purposes, a few design considerations are summarized as shown in Table 5. 

### 5.2. Coil Sensitivity

The sensitivity of a MIS pH probe is defined as the ability of the system to correctly determine the pH and evaluate the acidosis condition of a fetus. It is evaluated based on the signal produced at the Rx coil, which is known as the induced voltage. The induced voltage depends on the coil materials, coil structure or shape, coil core, coil arrangement, coil cross-sectional area, coil length and number of turns, coil winding, and insulation. In addition, voltage also depends on the applied frequency and the excitation current or voltage. The induced voltage measured at the Rx coil is also affected by noise from the surroundings.

The ability to obtain good sensitivity to a signal is called selectivity. A sensitive system would be able to identify a signal without being influenced too much by other properties in the sample that have changed over time. A system’s selectivity can be improved by expanding its measurement range and employing more advanced methods of extracting and connecting relevant data [50]. 

Signal resolution is based on the possibility of achieving a noise floor. A noise floor is a measurement of signal from the summation of all noise sources and unwanted signals within a system. Signal resolution improves with a smaller noise floor level [36,55]. The resolution of the coil sensor is limited by thermal noise, $V_{T}$ which depends on the resistance *R* of the coil, the temperature *T*, and the frequency bandwidth, ∆f with a coefficient equal to the Boltzmann factor $k_{B}$ = 1.38 × $10^{-23}$ Ws $K^{-1}$ [35].

(22) $$V_{T}=2\sqrt{k_{B}\;\cdot T\cdot ∆f\cdot R}$$

(23) $$R=\frac{\rho \cdot l}{A}$$

*R* depends on the resistivity, ρ length, *l*, and area of the coil, *A*. Increasing the coil length is not practical since sensitivity improves with length, while SNR only improves by $\sqrt{l}$ [35]. The best way to obtain maximum sensitivity and resolution is to increase the coil diameter *D* and increase the number of turns. The higher the number of magnetic fields penetrating areas of the coil, the greater the induced voltage in the Rx coil and the sensitivity is improved. 

In designing the MIS probe, obtaining a high signal-to-noise ratio is becoming one of the main objectives as noise sources limit the device’s sensitivity and accuracy. SNR quantifies the comparison of the signal received at Rx between the intended diagnostic signal and the noise signal measured using their amplitude [83]. A few main noise sources in MIS are temperature variations, lift-off, changes in EM properties (permeability and conductivity), additional magnetic noise (Barkhausen noise) [35], and changes in test speed [32]. 

There are a few methods that can be used to maximize SNR as listed below [32]. The first step is to amplify the amplitude level of the Rx signal. This might also amplify the noise, so the number of amplifications should be adjusted below the limit that depends on the design. High resolution of the analog to digital converter (ADC) is also required to improve the detection of dynamic range signals and SNR [39]. Next, filtering can be applied if the perturbation is not in the pass band of the desired signal. Phase discrimination techniques are used when there is a phase difference between the measured sample and the noise source. In addition, improvement can be made by changing the excitation modes [83]. 

Furthermore, some types of probe coil configurations are less influenced by noise; for example, self-compensated differential coil probes. Magnetic shielding can reduce the Rx noise obtained from the external sources, therefore increasing SNR. SNR can also be improved by selecting the most suitable sensor for the detection of specific properties of a specific sample. Different types of sensors are known to have their own specific sensitivity and noise level limits. For very low magnetic field applications, superconducting quantum interference devices (SQUID) can be used [32]. Gradiometers are useful, but high-order gradiometers can reduce the sensitivity and SNR ratio [35].

### 5.3. Coil Fabrication

There are various types of coil fabrication available; however, only photolithography, printed circuit boards and 3D printing techniques are discussed.

Photolitography provides a turn/space less than 20 µm in width (down to a few µm) which is very small in diameter and thickness. Since the copper thickness is reduced, the resistance of the coil is rapidly increased. In addition, the inductance will also increase as the turns will be closer to each other. Another disadvantage is the high price and availability for single-layer or double-layer planar coil only [39].

The printed circuit board (PCB) method has a minimal dimension of the turn/space of 100 µm and the drilled hole diameter is also 100 µm, which is five times bigger compared to the photolithography method. The PCB used for coil fabrication has advantages in terms of simplicity, low cost, good repeatability, uniform cross-section, and can fabricate more than 10 layers of planar coil [69]. On top of that, it is mechanically stable, has high consistency in fabrication, and it is easy to adjust the separation [39].

Additive 3D manufacturing or 3D printing technology allows for custom induction coil geometry design in the form of either a planar or non-planar PCB. It gives designers significant freedom over form factors to create new products and experiment with novel geometry. Coil is directly printed layer-by-layer on a substrate from nanoparticle conductive inks. The process thus allows for the embedding of specific inductor materials inside the substrate if desired during fabrication. Moreover, it can print custom tuning switches, impedance matching networks, and RF filters. This technology allows adjustment of the PCB to the specific probe geometry with the objective of saving space on the board, resulting in sleeker and smaller finished products. The greatly reduced fabrication and assembly time from weeks to hours expedites the process of successive design, build, and test iterations.

In comparison to traditional injection molding, casting, and PCB fabrication, 3D printing has lower material costs, shorter fabrication times, and fewer assembly steps. Besides, fabrication of EM coil probes using 3D printing anticipates high-complexity designs with a low-volume product. Nevertheless, the thin-film technology was reported for a flat planar micro-coil sensor application with <1 mm dimensions connected to an on-chip CMOS electronic circuit [35].

### 5.4. MIS Probe Testing

Simulation and in vitro techniques are very useful in assessing the MIS probe functionality. 

COMSOL Multiphysics, ANSYS Maxwell and MATLAB are software that show a great ability to solve Maxwell’s Equation by using the finite element method (FEM). In order to simplify the analysis, the following assumptions are made: (1) for the simulation, EM propagation is performed in three dimensions; (2) EM waves interact directly with the fetus’ head in an open region, which is air; (3) the dielectric properties of tissues are constant and uniform; (4) assume only blood exists, and increase design complexity by introducing other tissues into scalp layers and making comparisons.

The simulation involves the design of the real size of the MIS coil sensor probe (e.g., shape, diameter, turns, excitation) and the fetus scalp tissue model. Then, an ensemble model of the probe and tissue system enclosed in an air box with an adaptive mesh scheme is created. The high mesh resolution setting improves the accuracy, but it also increases the computational time. The solution is generated using the specified mesh setting. The analysis can be made from the magnetic field, impedance, induced voltage, and other properties found in Maxwell’s study solution. Study [54] have simulated an open-loop sensing coil design for both air-cored and ferrite-cored gradiometers and observed the induced current density as well as the induced voltage produced.

**Figure 22:**
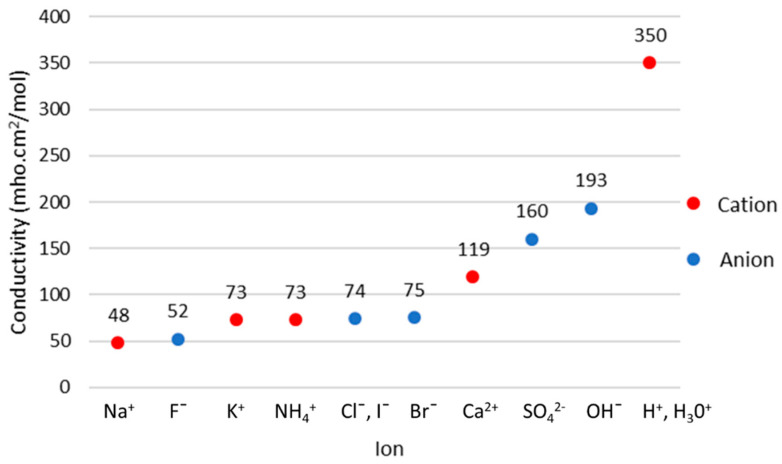
Molar conductivity of ions (cations and anions) [45].

In an experimental study, in vitro testing has previously been performed using a salt solution of sodium chloride (NaCl). NaCl was chosen as a weak acid ionic solution that resembles an acidosis condition with a pH range of 7.20–7.40 [65]. This is because these species, sodium (Na) and chlorine (Cl), were found to be the most dominant ions in human tissue fluids [45]. The concentrations for both of them are around 150 millimol/L, which is equivalent to 0.9 wt% saline solution. The conductivity of an electrolyte can be found from equation

(24) $$\sigma =\underset{i}{\sum }m_{i}\sigma_{m_{i}}$$

where m is the molar concentration of ion species with molar conductivity $\sigma_{m_{i}}$. Figure 22 shows the molar conductivity of ions in dilute aqueous solution at 25 °C. The highest conductivity is obtained by $H^{+}$, which is closely related to the measurement of pH value. Thus, implementing the MIS technique by measuring the conductivity could estimate the pH. However, pH measurement may be affected by the conductivity of other ions such as ${\mathrm{Na}}^{+}$ and $K^{+}$ which coexist in the tissue.

The performance of MIS was tested in the range of 0.01–10 S/m for low-conductivity objects. NaCl solutions were filled in 50 mL plastic bottles with a bottom thickness of 2 mm and placed close to the coil sensor. The experiment was conducted at an ambient temperature of 20–25 °C [39,54]. All sample measurements were repeated 10 times with <1.0 °C variance during each measurement. The conductivity of NaCl samples was compared to the commercial conductivity meter. It can also be compared to the established blood pH with their respective conductivity values shown in Table 6. The efficacy of the electric field measurements was obtained by determining the offset measurements of the distilled water and saline solutions [39,54]. 

The effect of conductivity on the distance was also studied where measurement was taken for a different distance from the sample to the Tx coil as the effect of lift-off [54]. The cell constant—that is, the parameter that relates the measured capacitance and conductance to the permittivity and conductivity of the sample—was obtained experimentally.

Phantom simulated data are commonly used to study the feasibility and capability of MIS systems in both biomedical and industrial applications. The advantage of using phantoms is that it is easy to develop different sizes, shapes, and conductivity of phantoms at a relatively low cost. Furthermore, the conductivity distribution of the phantoms can be kept uniform and free from contamination for a long period [79]. In this respect, simulation results may be easily validated against experimental data. However, phantom-based studies are limited by the scope of static or quasi-static conditions with a low frame rate (usually absent of real-time data) [79].

In a previous study, [84] has established a realistically physical head phantom. It requires modeling of head compartments with a well-defined volume conduction configuration and characterization of electrical conductivity. This phantom needs a synthetic material that is mechanically and electrochemically stable and possesses conductivity values similar to the modeled human head tissues. It has been concluded that agarose, gypsum, and NaCl solution can serve as stable representations of the three main conductivity compartments of the head, i.e., scalp (0.137 S/m to 2.1 S/m), skull (0.066 S/m and 0.00275 S/m), and intracranial volume (average of 0.33 S/m) [84]. 

NaCl is suggested to be the sample for MIS acidosis detection as it is easy to prepare and has a homogeneous characteristic. Next, the MIS pH probe can be tested using a real blood sample at 37 °C. However, blood coagulation should be avoided, which may affect the measurement of pH. For further analysis of MIS pH probe sensitivity, the model of multilayer scalp tissue can be developed using agarose and gypsum for in vitro testing. For in vivo testing, involving animal testing, all the experimental protocols should be approved and the care of animals should be carried out in accordance with the Declaration of Helsinki and International Association for the Study of Pain (IASP) guidelines [85]. 

### 5.5. Electronic Circuit

The MIS system consists of several components, which are sensors (Tx and Rx), an electronics interface, and a host computer [3]. The excitation circuit is simply connected to the function generator and power supply. At the Rx coil, an additional circuit is required in order to obtain the signal with high sensitivity. The Rx output signal can be connected to an integrator circuit or current-to-voltage converter [35]. Since biological signals are small, amplification is required using an operational amplifier that enlarges the differential signal. In order to remove noise, a second-order Butterworth low-pass filter with a specific cut-off frequency is further used as an anti-aliasing filter. The load impedance was found to increase proportionally with frequency since the coils work as inductors [39]. By implementing a large coil sensor and a sensitive amplifier, low biological tissue signals can be measured.

Furthermore, SNR can be increased effectively by using data filtering to eliminate or suppress noise at some frequency bands by applying a high-pass, low-pass, band-pass, or notch filter—although, for an intended signal with an overlapping frequency to the noise signal, filtering should not be applied to prevent elimination of informative signals. The filtering process in general follows the principle of Nyquist sampling and a Notch filter. Nyquist sampling was set to a sampling frequency at least two times the highest frequency of interest ($f_{sampling}\geq 2f_{signal}$) to avoid information loss. The Notch filter is useful to remove narrow-frequency noise such as power line interference (50 or 60 Hz) and the harmonics [72].

The output signal can also be improved by using a feedback loop. The feedback loop works by canceling out drifting imbalances due to harmonic distortions, thermal influences, and unwanted environmental noise with a coherent frequency. It is a simple yet effective and very low-cost approach [50]. The Rx coil signal can be connected to an integrator circuit or current-to-voltage transducer. For a multi-frequency input waveform, an ADC/DAC-FPGA is used to process the significant frequency components. The system receives amplified signals, filters the signal, provides signal compensation for auto-nulling, and returns frequency components to the host PC. The system has the capability to process the sample every 1.5 s approximately [50]. 

An important reminder is to warm up the electronic circuit system for 15 min before taking the measurement to obtain a stable measurement. This is because as the circuit is powered on, the temperature of electronic components increases gradually and requires a little time to reach a balanced state. This thermal imbalance may affect the measurement of the MIS system [39].

### 5.6. Analysis Method

MIS techniques have successfully observed pH changes in tissue [22]. They can be described in terms of induced magnetic field, induced current density, blood pH conductivity, phase change, and voltage. Anaerobic cellular respiration causes an increase in $H^{+}$ in the blood, thus increasing the conductivity of the blood [1]. Increasing the $H^{+}$ concentration also creates an acidic condition in the tissue and lowers the pH. Therefore, an increase in pH value causes a decrease in both the magnetic field and conductivity [19]. This proves that magnetic fields and conductivity are inversely proportional to pH values [65]. 

**Figure 23:**
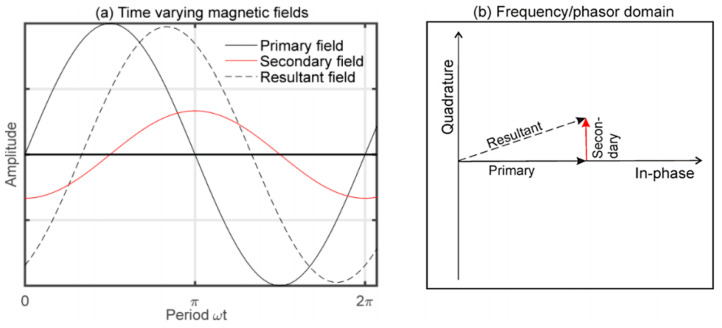
(**a**) Superimpose of primary, secondary and resultant magnetic field in time domain (**b**) Oscillating field represents by phasor line (in-phase and quadrature component) in frequency domain [78].

The phase-shift approach is an established method used in MIS measurement. Measurement of phase shift gives insights into the dielectric properties of biological tissue. The phase-shift method was previously implemented in the diagnosis of human diseases such as thermal injury. It is also able to detect edema tissue formation through measurement of large scale tissues in time and within a wide frequency range [6]. Phase delay from primary and secondary fields has been observed when the signal flows across the biological tissue [53], as shown in Figure 23a,b. This is because in biological tissues, magnetic skin depth is always large compared to the thickness of the scalp, so the secondary field is nearly 90° phase shifted with respect to the primary field [36].

The phase shift value obtained at Rx refers to the tissue properties: conductivity and permittivity. The conductivity increases with frequency due to an increasing magnetic field. A high excitation frequency given at the Tx coil will be able to generate a stronger primary magnetic field across the sample. Thus, it induces a higher eddy current and finally gives rise to a stronger secondary magnetic field which will be detected in the Rx coil [53]. Therefore, the magnitude of the magnetic field, ∆*B*, was normally found to be proportional to the conductivity of the sample and the frequency of the system [46].

The eddy current produced depends on the object conductivity. By applying electrical fields over a broad frequency range (1–10 MHz), complete dielectric dispersion properties for blood samples can be obtained; 10 MHz was chosen as the best frequency because it is in the range of biomedical tissue, which is good in current path when passing through biological tissue, and could provide a big enough field for conductivity of the target sample [46,65]. In addition, this frequency is also good enough to obtain the whole measurement system, which requires phase measurement accuracy of at least 0.01° [65]. Low frequency is used to compensate for the biological tissue characteristics of paramagnetic material and to maximize the EC flow at the surface of a sample [32].

**Figure 24:**
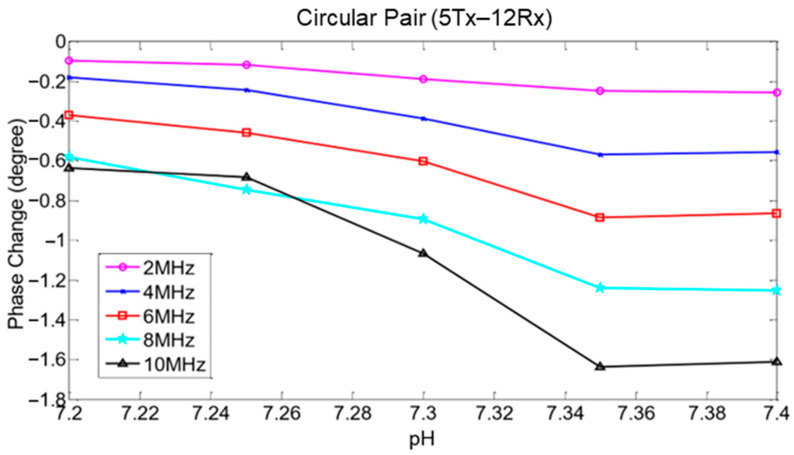
Phase change versus pH values for different frequencies for detection of acidosis [65]. “Adapted with permission from Ref. [65], 2017, Sarkawi, S. et al.”.

**Figure 25:**
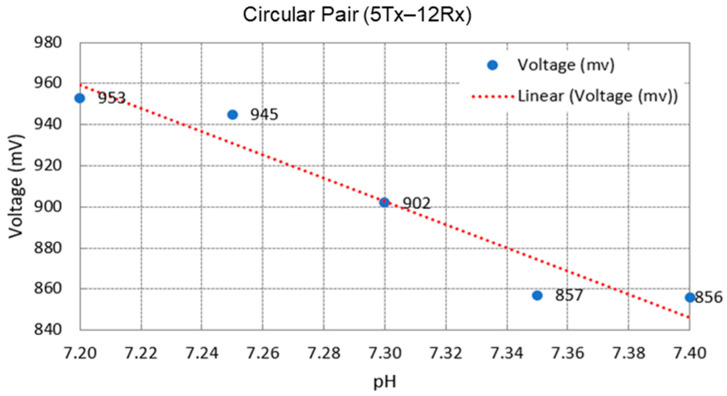
Linear regression line of voltage versus pH for normal and borderline (pre-acidosis) stage. “Adapted with permission from Ref. [65], 2017, Sarkawi, S. et al.”.

The phase shift versus pH plot is shown in Figure 24, where phase shift decreases as pH increases. The highest difference in phase shift at an instantaneous pH is observed for 10 MHz [22]. Therefore, 10 MHz was used to plot voltage versus pH (Figure 25) by applying formula

(25) $$V_{out}=\frac{G-90}{0.01}$$

where $V_{out}$ is the output voltage, *G* is the phase shift minus 90° phase shift, and 0.01 is the attenuation. This result is supported by the magnetic field versus pH plot which is also found to decrease as the pH value increases [19]. The Rx signal sensitivity is defined as the slope of the fitted straight line, which varies with pH. The slope, *S*, represents the sensitivity [54] of the system given S=∆V/∆pH. A steeper slope has been observed with an increasing excitation frequency (Figure 24). The optimum frequency is needed to reach the maximum sensitivity of the probe. 

Figure 25 shows the regression line that provides an equation for solving any given voltage to estimate pH. A linear regression model was applied to evaluate the linear relationship between voltage and pH (Equation (26)). The correlation coefficient ($R^{2}$) of the regression model indicates how close the data measured to the regression line (Equation (27)) [39]. A line-of-the-best-fit can be obtained by using the least-square method. High-sensitivity but low-linearity data should be excluded. The standard deviations at lower frequencies are larger due to low conductivity [50]. Linear regression [78] is the most suitable method as it correlates the changes in pH with the associated voltage. The model becomes a reference to approximate the scalp pH at an instantaneous voltage and vice versa. Thus, the condition of the fetus is either normal, borderline, or acidosis.

(26) $$y_{\mathrm{predicted}}=-569.8x+5062.2$$

(27) $$R^{2}=0.9058$$

The MIS pH result has been compared to a typical pH electrode response. A typical pH meter converts the voltage ratio between a reference half-cell and a sensing half-cell immersed in a solution with a known $H^{+}$ value. The reference half-cell is made up of silver or silver chloride electrode while the sensing half-cell is made up of a conductor immersed in a buffered electrolyte solution in conductive glass membrane sealed with epoxy tube. The pH meter shows that voltage changes linearly in relationship to pH changes. An increase in temperature will increase the voltage and also the slope of the graph. The reference for all standard voltage and temperature calibration is at 25 °C, where one pH unit corresponds to 59.16 mV as indicated by $H^{+}$ activity (*n* = 1).

The slope is defined by the Nernst factor as “2.3RT/nF”. The Nernst factor provides an amount of potential energy change for every 10-fold change in ion concentration. This means that for every pH unit change, the total potential will change by 59.16 mV as follows:

(28) $$E=E_{0}+2.3\frac{RT}{nF}\log\left(a_{i}\right)\;$$

where *E* is the potential (mV) between two reference and sensing half-cell, $E_{0}$ is standard potential of an ion, *R* is gas constant (J), *T* is absolute temperature (K), *n* is the ion’s charge, F is the Faraday constant (C/mol) and $a_{i}$ is the ion activity. The MIS pH probe can be compared to the typical pH meter; however, the resolution should be increased up to 0.01 pH changes as borderline of acidosis occurs in a very small range (7.20–7.25).

## 6. Conclusions

Monitoring of fetal condition during labor is crucial and could save a life. It is believed that MIS has a high potential to be a novel assessment tool for the fetal acidosis monitoring system. MIS pH meters allow users to measure blood pH quickly and non-invasively, which is designed for fetuses and neonates. Such a technique would certainly be useful in emergency medicine, critical care, and other clinical applications. In addition, the measuring device is small and portable.

This paper presents an overview of potential MIS in fetal acidosis detection and provides evidential basis for future development of MIS. The two major challenges of this device are the relatively small value of the secondary magnetic field and the existence of multi-layered scalp tissue with different dielectric properties. In our opinion, a successful design of the MIS pH probe requires an experimentally verifiable model with repeatable and stable measurements, such as other pH indicators. In this respect, it is recommended to have a number of benchmarks for the MIS test to be adapted by the research community for the validation purpose of various models. This requires more research for the probe system design in order to fulfil the requirements of sensitivity, specificity, reliability, and clinical validity. 

Future reviews should focus more on the real-world problems as sources of errors in MIS designs, for example, variation in fetal scalp thickness, body movements, and unspecified physiological changes such as temperature. In addition, a review of the electronic circuit for detection of micro to nano-amplitude signals is also needed. Nano technology in electronics could enhance biosignal detection, thus improving the overall MIS system. 

It is hoped that the development of the MIS pH probe will attract more attention from researchers in this field. Development of a millimeter-sized, contactless pH meter using magnetic sensors is believed to be able to substitute invasive fetal blood sampling in the near future. The MIS pH probe is expected to be commercialized as a rapid and safe diagnostic device for fetal acidosis detection, thereby contributing to both the public and social economy.

A list of nomenclature used throughout this review paper is provided in Table 7 as follows.

## Figures and Tables

**Table 1:** pH measurement for acidosis [1].

Measurands		Interpretation
pH	Lactate	
≥7.25	≤4.1 mmol/L	Normal
7.21–7.24	4.2–4.8 mmol/L	Borderline
≤7.20	≥4.9 mmol/L	Abnormal

**Table 2:** Tissue thickness in millimeter.

Type of Tissue	[59]	[60]	[61]	[62]
Skin	2.0	2.0	1.0	2.8
Fat	1.0	2.0	1.4	2.0
Muscle	4.0	2.0	-	1.7
Skull	10.0	5.2–8.5	6.6	5.5
Dura	1.0	0.5	-	-
CSF	2.0	4.9–7.9	-	1.5

**Table 3:** Permittivity and conductivity of skin, blood, fat, muscle, and skull for 1–10 MHz. “Adapted with permission from Ref. [63], 2021, https://itis.swiss/virtual-population/tissue-properties/database/dielectric-properties/ accessed on 10 August 2021”.

Frequency (MHz)	*ε_r_* Skin	σ Skin	*ε_r_* Blood	σ Blood	*ε_r_* Fat	σ Fat	*ε_r_* Muscle	σ Muscle	*ε_r_* Skull	σ Skull
1	9.91 × 10^2^	1.32 × 10^−2^	3.03 × 10^3^	8.22 × 10^−1^	5.08 × 10^1^	4.41 × 10^−2^	1.84 × 10^3^	5.03 × 10^−1^	2.49 × 10^2^	9.04 × 10^−2^
2	8.58 × 10^2^	3.71 × 10^−2^	1.68 × 10^3^	9.26 × 10^−1^	4.64 × 10^1^	4.48 × 10^−2^	8.26 × 10^2^	5.48 × 10^−1^	1.85 × 10^2^	9.71 × 10^−2^
3	7.46 × 10^2^	6.31 × 10^−2^	1.08 × 10^3^	9.83 × 10^−1^	4.36 × 10^1^	4.56 × 10^−2^	5.22 × 10^2^	5.68 × 10^−1^	1.48 × 10^2^	1.03 × 10^−1^
4	6.54 × 10^2^	8.82 × 10^−2^	7.73 × 10^2^	1.02 × 10	4.11 × 10^1^	4.66 × 10^−2^	3.85 × 10^2^	5.81 × 10^−1^	1.25 × 10^2^	1.07 × 10^−1^
5	5.79 × 10^2^	1.11 × 10^−1^	5.96× 10^2^	1.04 × 10	3.87 × 10^1^	4.77 × 10^−2^	3.08 × 10^2^	5.90 × 10^−1^	1.09 × 10^2^	1.11 × 10^−1^
6	5.18 × 10^2^	1.32 × 10^−1^	4.84× 10^2^	1.06 × 10	3.66 × 10^1^	4.88 × 10^−2^	2.60 × 10^2^	5.97 × 10^−1^	9.71 × 10^1^	1.14 × 10^−1^
7	4.68 × 10^2^	1.51 × 10^−1^	4.07× 10^2^	1.07 × 10	3.46 × 10^1^	4.98 × 10^−2^	2.27 × 10^2^	6.03 × 10^−1^	8.81 × 10^1^	1.16 × 10^−1^
8	4.26 × 10^2^	1.68 × 10^−1^	3.52× 10^2^	1.08 × 10	3.27 × 10^1^	5.08 × 10^−2^	2.03 × 10^2^	6.08 × 10^−1^	8.11 × 10^1^	1.19 × 10^−1^
9	3.91 × 10^2^	1.83 × 10^−1^	3.11× 10^2^	1.09 × 10	3.11 × 10^1^	5.17 × 10^−2^	1.85 × 10^2^	6.13 × 10^−1^	7.54 × 10^1^	1.21 × 10^−1^
10	3.62 × 10^2^	1.97 × 10^−1^	2.80× 10^2^	1.10 × 10	2.96 × 10^1^	5.26 × 10^−2^	1.71 × 10^2^	6.17 × 10^−1^	7.08 × 10^1^	1.23 × 10^−1^

**Table 4:** Dielectric properties of tissues for adult and 7-year-old children. “Adapted with permission from Ref. [66], 2018, Bhargava, D. et al.”.

Types of Tissues	Adult		7 Years Old	
	*ε* *_r_*	*σ* (*s*/*m*)	*ε* *_r_*	*σ* (*s*/*m*)
Skin	41.41	0.87	42.47	0.89
Fat	11.33	0.11	12.29	0.12
Bone	20.79	0.34	21.97	0.36
Brain	45.80	0.76	46.75	0.78
Eyes	36.59	0.51	49.60	0.99

**Table 5:** MIS probe design considerations for fetal acidosis detection [1].

Parameter	Functionality
Dimension	Probe diameter ≤ 1 cm should affix the cervix as it dilates from 1–10 cm. Probe length ≈ 10 cm in order to reach the fetal scalp located 2–3 cm [54] from the cervix opening before it dilates to allow fetus to move through the vagina during term.
Biocompatibility	Materials used should be biocompatible, e.g., ABS/Silicon [43]. Any chemical sensing must be affixed to ensure they are always attached to the sensor surface. Any chemicals used for sensing must be affixed in a way to ensure that they do not separate from the sensor surface. The probe materials must not degrade during sterilization
Accuracy and functionality	Electromagnetic compatibility (EMC) or no interference to other existing devices (e.g., CTG) Device should accomplish a continuous sampling rate <5 min interval and minimum 12 h measurement of measurands. Able to maintain pH reading with presence of: Vaginal secretions (pH 3.8–4.5); Amniotic fluid (pH 7.1–7.3); Various scalp tissue layers including skin, blood, fat, muscle, and skull with different conductivity and thickness; Variation in tissue layers properties for different fetuses; Temperature 35–42 °C; Fetus movement.
Safe operation of medical equipment	Comply the latest standard IEC 60601-1 (4th Edition) for extensive use in different global regions. 2nd Edition: Categories of increasing severity: Type B equipment 3rd Edition: Means of protection (MOP): Double isolation. Analyzing risk: Risk Management Process described in ISO 14971 Edition 3.1: Addressing 3rd Edition Ambiguities. 4th Edition: Electromagnetic disturbance (EMC concerns, IEC 60601-1-2). Intended used environments: Professional healthcare facilities. Current ≤1 A comply with FDA for medical devices. Leakage currents below 500 µA. Frequency ≤10 MHz is most suitable as indicated by Industrial, Scientific, and Medical (ISM) frequency bands [82]. Low Power. Patient vicinity, distance to the scalp (1–5 cm). Lift-off for short range wireless medical devices. Exposure time (1–2 s).
Coil design	Type of coil circular/square. Material copper/gold nanoparticle. Number of turns ratio, N for Tx < 2Rx. Specify inner and outer diameter of Tx and Rx coil. Specify distance between Tx-Rx coil. Arrangement of Tx-Rx coil; planar/gradiometer/perpendicular. Excitation using single/multifrequency.

**Table 6:** Conductivity for different blood pH. “Adapted with permission from Ref. [19], 2016, Sarkawi, S. et al.”.

**pH**	6.5	6.6	6.7	6.8	6.9	7.0	7.1	7.2	7.3	7.4
**σ (S/m)**	1.9	1.8	1.7	1.6	1.5	1.4	1.3	1.2	1.1	1.0

**Table 7:** List of nomenclature used in this review paper.

Nomenclature	Referred to	Nomenclature	Referred to
B	Magnetic Field Density	Rx	Receiver coil
C	Capacitance	SNR	Signal to Noise Ratio
CTG	Cardiotocography	Tx	Excitation coil
EC	Eddy Current	V	Voltage
ECG	Electrocardiogram	H^+^	Hydrogen ion
EMF	Electromotive Force	CO_2_	Carbon dioxide
FBS	Fetal Blood Sampling	H_2_CO_3_	Carbonic acid
FHR	Fetal Heart Rate	HCO_3_^−^	Bicarbonate ion
H	Magnetic Field Strength	*ε*	Permittivity
MIS	Magnetic Induction Spectroscopy	*σ*	Conductivity
NaCl	Sodium Chloride	*μ*	Permeability
PCB	Printed Circuit Board	*ϕ*	Magnetic flux
R	Resistance		

## Data Availability

No new data were created or analyzed in this study. Data sharing is not applicable to this article.
